# Research on the Bearing Remaining Useful Life Prediction Method Based on Optimized BiLSTM

**DOI:** 10.3390/s25144351

**Published:** 2025-07-11

**Authors:** Yi Zou, Wenlei Sun, Tiantian Xu, Bingkai Wang

**Affiliations:** School of Mechanical Engineering, Xinjiang University, Urumqi 830047, China; 107556521219@stu.xju.edu.cn (Y.Z.);

**Keywords:** remaining useful life, optimized BiLSTM, bearing, threshold, health indicators

## Abstract

The predictive performance of the remaining useful life (RUL) estimation model for bearings is of utmost importance, and the setting method of the bearing degradation threshold is crucial for detecting its early degradation point, as it significantly affects the performance of the RUL prediction model. To solve these problems, a bearing RUL prediction method based on early degradation detection and optimized BiLSTM is proposed: an optimized VMD combined with the Pearson correlation coefficient is used to denoise the bearing signal. Afterward, multi-domain features are extracted and evaluated using different metrics. The optimal degradation feature is then selected. Furthermore, KPCA is used to integrate the features and establish the health indicators (HIs) for early degradation detection of bearings using a sliding window method combined with the 3σ (3-sigma) criterion and the quartile method. The RUL prediction model is developed by combining the BiLSTM network with the attention mechanism and by employing the SSA to adaptively update the network parameters. The proposed RUL prediction model is tested on various datasets to evaluate its generalization ability and applicability. The obtained results demonstrate that the proposed denoising method has high performance. The dynamic 3σ-threshold setting method accurately detects the early degradation points of bearings. The proposed RUL prediction model has high performance and fitting capacity, as well as very high generalization ability and applicability, enabling the early prediction of bearing RUL.

## 1. Introduction

With the advent of Industry 4.0, the rotating machinery is gradually evolving toward intelligence, while its operating conditions became complex; therefore, ensuring safe and effective operation is a basic requirement of modern society [1]. Bearings are a key component that supports the rotating shaft in rotating machinery. The accurate prediction of the RUL of bearings allows a reduction in maintenance costs, improves the economic benefits, and prevents human and material losses caused by unexpected machine shutdowns [2,3]. The commonly used RUL prediction models can be divided into four types: physics-based, expert knowledge-based, hybrid method-based, and data-driven [4]. The physics-based prediction methods develop physical mechanism models of bearing failure. They adopt the Lundberg–Palmgren theory to calculate the bearing fatigue life through stress–life (S-N) curves, which is suitable for evaluating the life of different design schemes in the early stages of bearing design. They are directly based on the physical characteristics of the bearing failure, with strong theoretical explicability. However, they exhibit complex modeling and high computational load [5]. The expert knowledge-based prediction methods encode typical cases of bearing failure, expert experience, rule bases, and other knowledge into logical rules through expert systems or knowledge graphs. They also adopt real-time monitoring data to infer the life state of bearings. They have high flexibility without the need for large amounts of historical data. However, they are highly subjective, and updating their rules is slow [6]. The hybrid method-based approach to prediction leverages the advantages of physical models and data-driven methods to develop hybrid prediction models. It has high adaptability and generalization ability. However, its parameter optimization is difficult and it has a high computational load [7]. The data-driven prediction methods use machine learning or deep learning algorithms for feature extraction and modeling. They have high accuracy, real-time performance, and adaptability. A large number of studies have started to use data-driven methods for predicting the RUL of bearings [8,9,10,11]. At present, data-driven technologies mainly include CNN [12], long short-term memory (LSTM) and its variants [13], Gated Recurrent Units (GRUs) and their variants [14], RNNs and their variants [15]. The faults generated during the operation of bearings are inherently difficult to observe. Therefore, it is crucial to establish an HI in their degradation process, perform early degradation point detection, and develop RUL prediction models. Although the original vibration signals contain rich fault information, traditional single-feature extraction methods cannot comprehensively capture all fault features. This leads to incomplete information extraction, which affects the accuracy of bearing life prediction. Therefore, multi-feature fusion methods have emerged. They are able to comprehensively capture fault information in bearing vibration signals, and thus they allow for better prediction of the bearing life [16]. Zhang et al. [17] combined a TCN and an LSTM network to establish a multi-branch network based on parallel feature extraction. This method allows us to perform adaptive feature weighting and fusion, in order to accurately predict the remaining useful life (RUL) of bearings. Liu et al. [18] combined the time-domain data of bearing vibration signals and performance degradation data and used a Preprocessor–LSTM approach to predict the RUL of bearings. Li et al. [19] designed a multi-branch MBCNN prediction network, which uses raw bearing vibration signals and frequency-domain signals as dual-branch inputs. They performed RUL prediction for bearings through feature prediction and fusion. Mi et al. [20] solved the problem of insufficient feature selection and fusion for bearings by proposing a two-layer feature selection method, allowing to increase the accuracy of RUL prediction. Zhao et al. [21] used signal processing and feature fusion techniques to establish a nonlinear health indicator (HI) for bearings. They then proposed a method combining vibration signals and physical models in order to reduce the errors of RUL prediction. Zhang et al. [22] evaluated the monotonicity, robustness, and fitting errors of bearing signals to develop a fusion model for RUL prediction through weighted fusion. Tai et al. [23] used KPCA and exponential weighted average methods to build a bearing HI. This approach allowed to accurately predict the HI and RUL of bearings. Yang et al. [24] highlighted the importance of early fault detection in predicting the RUL of bearings. In normal operation, the HI remains stable. However, when faults start to occur, it changes significantly, which makes the detection of early fault points critical. Wang et al. [25] used probabilistic models for RUL prediction and early fault detection methods for determining the initial fault point of bearings. Chen et al. [26] applied the 3σ criterion to detect early fault points in bearing signals. They also combined an adaptive Wiener process with GRU for RUL prediction. Li et al. [27] proposed methods for detecting the initial degradation point (IDP) and fast degradation point (FDP) of bearing signals, enabling RUL prediction. Wang et al. [28] detected early degradation points in bearings. Cui et al. [29] used a sliding window algorithm to update the relative error of RUL predictions, allowing the identification of the first change point of faults. Wang et al. [30] proposed a hybrid model to predict the degradation trend of bearings. In addition, they used the 3σ criterion to determine early fault points in the process of signal degradation. Han et al. [31] proposed a stacked autoencoder method to fuse bearing features for RUL prediction. Balamurugan et al. [32] proposed a CNN-BiLSTM-based bearing data monitoring method allowing the detection of current and historical abnormal data. This method adopts KPCA for dimensionality reduction and CNN-BiLSTM for RUL prediction. Shen et al. [33] selected redundant features of bearings through composite evaluation metrics, established nonlinear degradation indicators (NDI), and proposed an attention-based BiLSTM network. Their results showed that this method outperforms existing ones on various datasets. 

Although some studies proposed methods for processing bearing vibration signals and predicting the RUL, several limitations exist, especially the low denoising performance leading to low signal purity. The use of a single feature as HI for bearings, along with a fixed threshold for degradation detection, leads to delayed detection of early bearing degradation, which results in missed fault diagnoses. In addition, LSTM cannot process information in parallel, and BiLSTM requires waiting for the complete input sequence before making predictions. Based on the aforementioned shortcomings of RUL prediction, this paper proposes an RUL prediction method that optimizes the BiLSTM network using the SSA [34,35]. Firstly, the vibration signals of the bearings are preprocessed, multi-domain features are extracted and fused, a health indicator is constructed, and a failure threshold is set. Finally, RUL prediction is conducted to obtain more reliable RUL prediction results for the bearings. This paper is mainly divided into the following sections:

Section 1: a comprehensive review is conducted on the research background of the entire thesis. 

Section 2: The NGO [36] algorithm is used to optimize the VMD [37] algorithm. Together with the Pearson correlation coefficient, it is applied to denoise the original bearing signals. Its performance is finally compared with that of other denoising methods. Multi-domain features of the bearing are extracted, and a comprehensive weighted index, which includes robustness, predictability, trendability, and monotonicity, is applied to quantify the scores of each feature. 

Section 3: The optimal features are selected and fused using KPCA. A method that combines the 3σ criterion and a sliding window is used to dynamically update the degradation threshold of the bearing in real time, which allows the early detection of bearing degradation. A RUL model is established by combining BiLSTM with an attention mechanism, and the SSA is employed to automatically search for network parameters. Experimental validation is then conducted. 

Section 4: the proposed method is validated through experiments, followed by an in-depth analysis of various phenomena observed during the process. 

Section 5: the primary conclusions of the entire thesis are drawn. 

**Figure 1:**
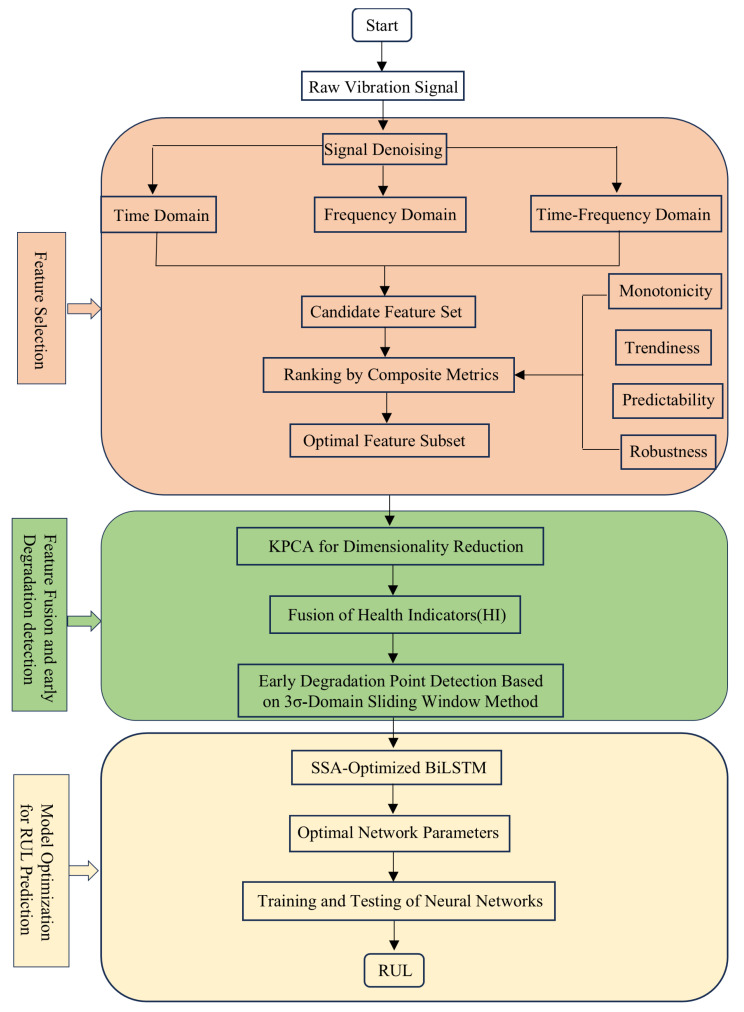
Overall technical roadmap.

The overall RUL prediction technical roadmap is shown in Figure 1. 

## 2. Preprocessing Methods for Bearing Vibration Signals

### 2.1. Signal Denoising Methods

The NGO algorithm is used to optimize the VMD algorithm. The optimized mode decomposition algorithm is referred to as NVMD. It consists of adaptively searching for the penalty factor (α) and the number of decomposed modes (k). The Pearson correlation coefficient (PCC) is used to measure the correlation between each mode and the original signal. The modes exhibiting weak (or no) correlation are considered noise (or irrelevant signals), which improves the signal purity. Afterward, each mode is reconstructed, and the results are compared with those obtained by the soft-threshold denoising method. The performance is evaluated using the Noise Mode (NM) and Signal-to-Noise Ratio (SNR) metrics. 

#### 2.1.1. Variational Mode Decomposition (VMD) Algorithm

VMD solves a constrained variational problem to decompose the input signal *f* into *k* discrete sub-modes $u_{k_{0}}$. This can be expressed as follows [36]:

(1) $$\underset{\left\{u_{{\,}_{k_{0}}}\right\},\left\{\omega_{{\,}_{k_{0}}}\right\}}{\min}\left\{{\sum_{k_{0}}\left\|\partial_{{\,}_{t_{0}}}[(\delta (t_{0})+\frac{j}{\pi t_{0}})*u_{k_{0}}(t_{0})e^{-j\omega_{k_{0}}t_{0}}\right\|}_{2}^{2}\right\}$$

(2) $$s.t_{0}.\sum_{k_{0}}u_{k_{0}}=f$$

where {$u_{k_{0}}$}: = {*u*_1_, ···, $u_{k_{0}}$} represents the shorthand for the modes obtained after signal decomposition, {$\omega_{k_{0}}$}: = {*ω*_1_, ···, $\omega_{k_{0}}$} represents the corresponding central frequencies, $\delta (t_{0})$ is the Dirac delta function, and $\sum_{k_{0}}\;:\;=\sum_{K_{0}=1}^{K_{0}}$ denotes the sum of all the underlying modes. 

In order to more easily solve the constrained variational problem, a quadratic penalty function term and a Lagrange multiplier λ are introduced to reformulate the problem as an unconstrained optimization one [36]:

(3) $$\begin{array}{l} \Gamma (\{u_{k_{0}}\},\{\omega_{k_{0}}\},\lambda )=\alpha {\sum_{k_{0}}\left\|\partial_{t_{0}}[(\delta (t_{0})+\frac{j}{\pi t_{0}})\cdot u_{k_{0}}(t_{0})]\cdot e^{-j\omega_{k_{0}}t_{0}}\right\|}_{2}^{2}\\ +{\left\|f(t_{0})-\sum_{k_{0}}u_{k_{0}}(t_{0})\right\|}_{2}^{2}+\langle \lambda (t_{0}),f(t_{0})-\sum_{k_{0}}u_{k_{0}}(t_{0})\rangle \end{array}$$

where multiplicative operators are used to iteratively update ${u_{k_{0}}}^{n+1}$, ${\omega_{k_{0}}}^{n+1}$, and $\lambda^{n+1}$ until the variational model reaches an optimal solution. The modal components can be expressed as follows [36]:

(4) $$\begin{array}{l} u_{k_{0}}^{n+1}=\arg\min\{\alpha {\sum_{k_{0}}\left\|\partial_{t_{0}}[(\delta (t_{0})+\frac{j}{\pi t_{0}})\cdot u_{k_{0}}(t_{0})]\cdot e^{-j\omega_{k_{0}}t_{0}}\right\|}_{2}^{2}\\ +{\left\|f(t_{0})-\sum_{i_{0}}u_{i_{o}}(t_{0})+\frac{\lambda (t_{0})}{2}\right\|}_{2}^{2}\} \end{array}$$

By applying the Parseval/Plancherel Fourier isometry transform, Equation (4) is mapped into the frequency domain [36]:

(5) $${\overset{\wedge }{u}}_{k_{0}}^{n+1}(\omega )=\frac{\overset{\wedge }{f}(\omega )-\sum_{i_{0}\neq k_{0}}\overset{\wedge }{u_{{\,}_{i_{0}}}}(\omega )+\frac{\overset{\wedge }{\lambda }(\omega )}{2}}{1+2\alpha {\left(\omega -\omega_{k_{0}}\right)}^{2}}$$

where $\hat{u_{k_{0}}{\,}^{n+1}}$ is the current residual of $\hat{f}(\omega )-\sum_{i_{0}\neq k_{0}}\hat{u_{i_{0}}}(\omega )$ after Wiener filtering. 

By continuously updating until the central frequencies are determined, we obtain the following [36]:

(6) $$\omega_{k_{0}}^{n+1}(\omega )=\frac{\int_{0}^{\infty }\omega {\left|\hat{u_{k_{0}}}(\omega )\right|}^{2}d\omega }{\int_{0}^{\infty }{\left|\hat{u_{k_{0}}}(\omega )\right|}^{2}d\omega }$$

where $\omega_{k_{0}}^{n+1}$ represents the centroid of the power spectrum of the current modal function. Note that the real part of the inverse Fourier transform of $\left\{u_{k_{0}}(\omega )\right\}$ yields $\left\{u_{k_{0}}(t_{0})\right\}$. 

#### 2.1.2. Optimizing Variational Mode Decomposition (VMD) Using the Northern Goshawk Optimization (NGO) Algorithm

The optimization of VMD using the Northern Goshawk Optimization (NGO) algorithm mainly consists of two stages. The first one involves prey identification and attack (i.e., exploration phase), where the Northern Goshawk randomly selects a prey and quickly attacks it. This corresponds to a random solution in the search space, which is updated by specific strategies in order to locate the optimal solution. This phase helps identify the optimal search area (i.e., the approximate position of the global optimum). 

The population is represented using the following population matrix [37]:

(7) $$X=[X_{1},X_{2},\ldots ,X_{N_{0}}]$$

where X*i*_1_(*i*_1_ = 1, 2, …, N_0_) represents the position of an individual. 

The objective function value is given by the following [37]:

(8) $$F=[F_{1},F_{2},\ldots ,F_{N_{0}}]$$

where *F* is the function vector, and *F_i_* is the objective function value for individual *i*. 

The determined optimal region is given by *P_i_*_1_ = *X_k1_*, *i*_1_ = 1, 2, ···, N_0_, *k*_1_ = 1, 2, ···, *i*_1_ − 1, *i_1_* + 1···, N_0_ [37].

(9) $$X_{i_{1},j_{1}}^{new,P1}=\{\begin{array}{c} x_{{\,}_{i_{1},j_{1}}}+r(p_{i_{1},j_{1}}-Ix_{{\,}_{i_{1},j_{1}}}),F_{P_{i}{\,}_{{\,}_{1}}}\geq F_{i_{1}}\\ x_{i_{1},j_{1}}+r(p_{i_{1},j_{1}}-Ix_{i_{1},j_{1}}),F_{P_{i}{\,}_{{\,}_{1}}}<F_{i_{1}} \end{array}$$

(10) $$X_{i_{1}}={\{}_{X_{i_{1}},F_{i_{1}}^{new,P1}\geq F_{i_{1}}}^{X_{i_{1},j_{1}}^{new,P1},F_{i_{1}}^{new,P1}<F_{i_{1}}}$$

where $P_{i}{\,}_{{\,}_{1}}$ is the prey position for the *i*1th Northern Goshawk, $F_{P_{i}{\,}_{{\,}_{1}}}$ is the objective function value, $X_{i_{1},j_{1}}^{new,P1}$ is its value in the *j*th dimension, and $F_{i_{1}}^{new,P1}$ is the first-phase objective function value.

The second stage consists of pursuit and escape (development stage). When the Northern Goshawk approaches its prey, the latter attempts to quickly escape, while the Northern Goshawk continues to pursue it. This corresponds to a detailed search near the optimal region to identify the most precise solution. This stage uses local search to increase optimization accuracy. 

In a region of radius *R*_0_, the target is searched for as follows [37]:

(11) $$x_{i_{2},j_{2}}^{new,P2}=x_{i_{2},j_{2}}+R_{0}(2r-1)x_{i_{2},j_{2}}$$

(12) $$R_{0}=0.02(1-\frac{a}{A})$$

(13) $$X_{i_{2}}={\{}_{X_{i_{2}},F_{i_{2}}^{new,P2}\geq F_{i_{2}}}^{X_{i_{2},j_{2}}^{new,P2},F_{i_{2}}^{new,P2}<F_{i_{2}}}$$

where *a* is the current iteration, *A* is the maximum number of iterations, $x_{i_{2},j_{2}}^{new,P2}$ is the value in the *j*th dimension, and $F_{i_{2}}^{new,P2}$ is the second-phase objective function value. *r* is a random number in the range of 0–1, *I* is equal to 1 or 2, and *r* and *I* are parameters for generating random NGO behaviors during search and update operations, respectively. 

#### 2.1.3. Pearson Correlation Coefficient

In the modal decomposition of bearing signals, the Pearson correlation coefficient [21] is used to measure the similarity between the original data E:{e_1_, e_2_, …, e*_na_*} and the modes B:{b_1_, b_2_, …, b*_na_*}. The correlation levels between arrays are in the range of 0.8–1. Very strong, strong, moderate, weak, and very weak (or no) correlation types are, respectively, represented by the ranges of 0.6–0.8, 0.4–0.6, 0.2–0.4, and 0.0–0.2 [21]. 

The overall mean and overall covariance are given by the following [21]:

(14) $$E(E)=\frac{\sum_{e_{n_{a}}=1}^{n_{a}}E_{n_{a}}}{n_{a}}$$

(15) $$E(B)=\frac{\sum_{b_{n_{a}}=1}^{n_{a}}B_{n_{a}}}{n_{a}}$$

(16) $$Cov(E,B)=\frac{\sum_{n_{a}=1}^{n_{a}}\left(E_{n_{a}}-E(E))(B_{n_{a}}-E(B)\right)}{n_{a}}$$

where *n_a_* represents the number of signal data points. 

#### 2.1.4. Denoising Evaluation Metrics

Noise Mode is given by [15]

(17) $$NM=\sqrt{\frac{1}{n_{c}}\sum_{t_{1}=1}^{n_{c}}{\left[(x(t_{1})-\hat{x(t_{1}})\right]}^{2}}$$

where *x*(*t*_1_) and $\hat{x(t_{1}})$ are the original and denoised signals, respectively, *n_c_* represents the number of signal data points, and *t*_1_ represents the time point. 

The Signal-to-Noise Ratio is calculated as [15]

(18) $$SNR=10\times \mathrm{lg}\left[\frac{\sum_{t_{1}=1}^{n}x^{2}(t_{1})}{\sum_{t_{1}=1}^{n_{c}}{\left(x(t_{1})-\overset{\wedge }{x(t_{1})}\right)}^{2}}\right]$$

### 2.2. Multi-Domain Feature Extraction and Domain Selection Method

Multi-domain feature extraction [36] can combine information from different domains to provide a more comprehensive description of the fault, thereby helping to improve the accuracy of the prediction model. By extracting and fusing multi-domain features, a more robust and accurate RUL prediction model can be constructed [23,24]. To extract richer information on bearing degradation, signals in the time [36], frequency [36], and time–frequency domains [36] are analyzed. The optimal features are first selected, and feature fusion is then performed. 

#### 2.2.1. Multi-Domain Feature Extraction

Table 1 presents the formulas for time-domain and frequency-domain feature calculations. Numbers 1–7 correspond to time-domain feature formulas, the unit for numbers 1, 2, and 6 is m/s^2^, that for numbers 3 and 5 is (m/s^2^)^2^, and numbers 4 and 7 are dimensionless. Time-domain features are characterized by changes in the signal waveform over time. Numbers 8–13 correspond to frequency-domain feature formulas. Numbers 8–11 are dimensionless, and the units of numbers 12 and 13 are m/s^2^ and Hz, respectively [38]. 

The db6 wavelet basis function is used for feature extraction in the time–frequency domain. A three-level wavelet packet decomposition is performed, yielding 2^3^ = 8 sub-frequency bands [36,37,38]. The wavelet energy entropy of these eight sub-frequency bands is calculated as follows [38]:

(19) $$WEE=-\sum_{i=1}^{8}\left(\frac{E_{i}}{E}\cdot \log(\frac{E_{i}}{E}\right))$$

where $E_{i}$ represents the energy characteristic of the *i*th sub-band, and *E* is the total energy of the eight sub-frequency bands [38]. 

The energy ratio of each sub-frequency band is calculated as [36,37,38]:

(20) $$P_{i}=\frac{E_{i}}{E}\times 100\%$$

There are 8 different energy ratios, denoted by p_14_(3,0), p_15_(3,1), p_16_(3,2), p_17_(3,3), p_18_(3,4), p_19_(3,5), p_20_(3,6), and p_21_(3,7). Note that these energy ratios are dimensionless. 

Based on the aforementioned analysis, 21 features should be extracted: 7 time-domain, 6 frequency-domain, and 8 time–frequency domain features [36,37,38]. 

#### 2.2.2. Feature Selection Method

The monotonicity, trendability, predictability, and robustness of each bearing signal feature are comprehensively evaluated. These four metrics are then quantified, and a comprehensive score is calculated. Note that a higher score indicates a higher sensitivity, more significantly contributing to bearing life prediction [37,38]. 

The monotonicity metric (*M*) is expressed as [37,38]

(21) $$M=\frac{1}{m}|\frac{positivediff(x_{c}^{n})-negativediff(x_{c}^{n})}{d-1}|$$

where *x_c_* represents the *c*th feature, *d* denotes the number of measurements for each one, and *m* is the number of monitored systems [37,38]. 

The trendability indicator (*Q*) is expressed as [37,38]

(22) $$Q=\frac{\left|\sum_{i_{b}=1}^{No}\left(x_{i}{\,}_{{\,}_{b}}-x_{m})(y_{i_{b}}-y_{m}\right)\right|}{\sqrt{\sum_{i_{b}=1}^{No}{\left(x_{i_{b}}-x_{m}\right)}^{2}\sum_{i_{b}=1}^{No}{\left(y_{i_{b}}-y_{m}\right)}^{2}}}$$

where *x*${{\,}_{i}}_{{\,}_{b}}$ is the rank of features, *y*${{\,}_{i}}_{{\,}_{b}}$ is the rank of time, and *N* is the number of feature measurements. Note that, for *Q* = 1, the feature is strictly monotonic, while for *Q* = 0, it is non-monotonic [37,38]. 

The predictability indicator (*C*) is expressed as [37,38]

(23) $$C=\exp(-\frac{std(f_{i_{c}}(N))}{mean|f_{i_{c}}(1)-f_{N_{c}}(N_{c})|})$$

where $f_{i_{c}}$ (1) and $f_{i_{c}}$ (*N_c_*) are the initial and failure values of the *i*_c_-th feature, respectively. 

The robustness indicator (*R*) is expressed as [37,38]

(24) $$R=\frac{1}{N_{R}}\sum_{i_{R}}\exp(-\frac{f_{i_{R}}-\tilde{f})}{f_{i_{R}}})$$

where $f_{i_{R}}$ is the *i_R_*-th feature, *N_R_* represents its measurement value, and $\tilde{f}$ is the average trend value [37,38]. 

The final weighted score is obtained by summing all the metrics [37,38]:

(25) $$ScoreJ_{j}=w^{\prime }{\,}_{1}M_{j}+w^{\prime }{\,}_{2}Q_{j}+w^{\prime }{\,}_{3}F_{j}+w^{\prime }{\,}_{4}R_{j}$$

where $w^{\prime }{\,}_{1}$, $w^{\prime }{\,}_{2}$, $w^{\prime }{\,}_{3}$ and $w^{\prime }{\,}_{4}$ are, respectively, the weights for the *M*, *Q*, *C*, and *R* metrics that are positive and satisfying $w^{\prime }{\,}_{1}+w^{\prime }{\,}_{2}+w^{\prime }{\,}_{3}+w^{\prime }{\,}_{4}=1$, $w^{\prime }{\,}_{1}$ > 0, $w^{\prime }{\,}_{2}$ > 0, $w^{\prime }{\,}_{3}$> 0, and $w^{\prime }{\,}_{4}$ > 0 [37,38]. The study presented in [38] shows that it is crucial to focus on the full life-cycle trend of the bearing and its tolerance to noise and abnormal signals. Therefore, when selecting features, greater emphasis should be placed on monotonicity and robustness. $w^{\prime }{\,}_{1}$, $w^{\prime }{\,}_{2}$, $w^{\prime }{\,}_{3}$, and $w^{\prime }{\,}_{4}$ are set to 0.4, 0.2, 0.1, and 0.3, respectively. Based on the results obtained in [20], features with a *J* score greater than 0.5 are selected. 

## 3. Method for Establishing RUL Prediction Model Based on Optimized BiLSTM Networks

### 3.1. Construction of Health Indicators and Determination of Failure Thresholds

#### 3.1.1. Construction of Health Indicators (HIs)

These features are fused using the KPCA method [23]. This process consists of the following steps:

(1) The sensitive feature subset is standardized, and the resultant feature vector matrix $\tilde{X}$ undergoes a nonlinear mapping using the function Φ, projecting $\tilde{X}$ to a higher-dimensional feature space ℜ. The samples in the input space are transformed via Φ, denoted as $\tilde{X}\to \Phi (X)$, and become sample points within the high-dimensional feature space ℜ, where $\Phi (X)=\left[\Phi (x_{1}),\Phi (x_{2}),\ldots ,\Phi (x_{n_{d}})\right]$, satisfying the centrality condition [27,38]:
  (26) $$\sum_{k=1}^{n}\Phi (x_{k})=0$$
  The covariance matrix in ℜ is given by $\bar{C}$ [38]:
  (27) $$\bar{C}=\frac{1}{n_{d}}\sum_{j_{0}=1}^{n}\Phi (x_{j_{0}}){\left[\Phi (x_{j_{0}})\right]}^{T}$$
  where $\Phi (x_{j_{0}})$ represents the feature sample in [38] ℜ.
(2) Calculate the eigenvalues and eigenvectors of the covariance matrix $\bar{C}$ [36,38]. The eigenvectors thus obtained represent the principal component directions of the original sample space in the feature space [37,38] ℜ:
  (28) $$\lambda v=\bar{C}v$$
  where λ represents the eigenvalue of $\bar{C}$, and *v* denotes the corresponding eigenvector in the feature space ℜ [37,38].
(3) Given the computational challenge of directly solving Φ(X), a kernel function $k(x_{i},x_{j})=\Phi {\left(x_{i}\right)}^{T}\Phi (x_{j})$ is introduced, resulting in an n×n symmetric positive definite matrix *K*, where *K* is the kernel matrix corresponding to $k(x_{i},x_{j})$, denoted as $K_{ij}=k(x_{i},x_{j})$ [23].
(4) Centering the kernel matrix *K* yields the standardized kernel matrix, represented by the following formula [23]:
  (29) $$\bar{K}=K-KI_{1/n}-I_{1/n}K+I_{1/n}KI_{1/n}$$
  where *I* is an n×n matrix where each element is 1/*n*.
(5) The eigenvalues and eigenvectors of $\bar{K}$, denoted as $\lambda_{1}^{\prime },\lambda^{\prime }{\,}_{2},\lambda_{3}^{\prime }\ldots ,\lambda^{\prime }{\,}_{n}$, $T_{1},T_{2},T_{3},\ldots ,T_{n}$, are calculated and arranged in descending order, represented by the following formula [23]:
  (30) $$\lambda^{\prime }T=\bar{K}T$$
(6) The eigenvectors $T_{1},T_{2},T_{3},\ldots ,T_{n}$ undergo Schmidt orthogonal normalization to produce the feature component matrix $\beta_{1},\beta_{2},\beta_{3}\ldots ,\beta_{n}$.
(7) Extract the eigenvalue of the first principal component that accounts for more than 85% [23] of the variance, along with its corresponding eigenvector $\beta_{1}$ to form the projection matrix *Y*, where $Y=\bar{K}\beta_{1}$ [23], and *Y* represents the fused feature derived from the original feature matrix *X* through Kernel Principal Component Analysis (KPCA) for dimensionality reduction, serving as a health indicator (HI).

#### 3.1.2. Determination of Failure Thresholds

**Figure 2:**
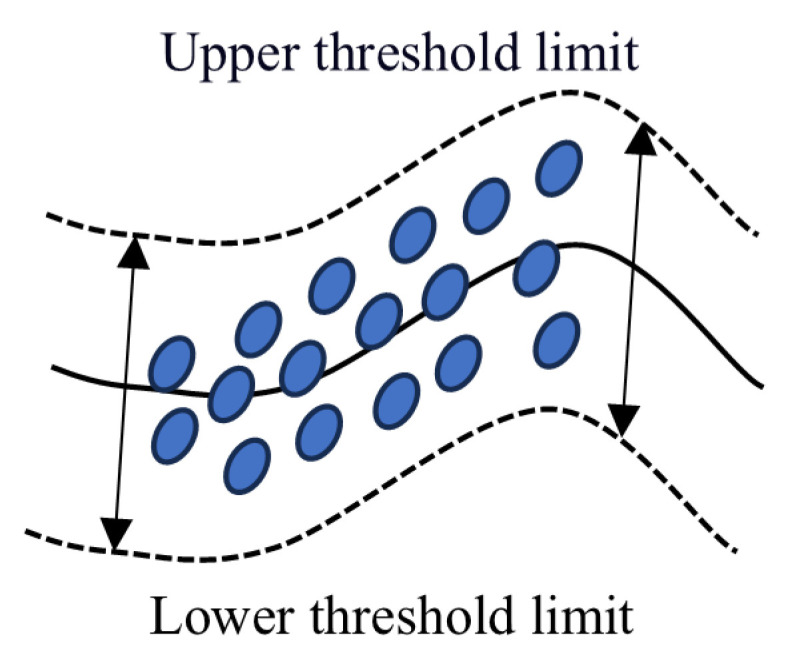
Schematic diagram of dynamic thresholds.

The signal is processed using a sliding window [30,31] approach. Within each window, the 3σ [26,32] method is combined with the quartile method [25] to identify significant change points indicative of fault occurrence. This combination reduces false positives and enhances detection accuracy. A schematic diagram of the dynamic threshold is shown in Figure 2.

**Figure 3:**
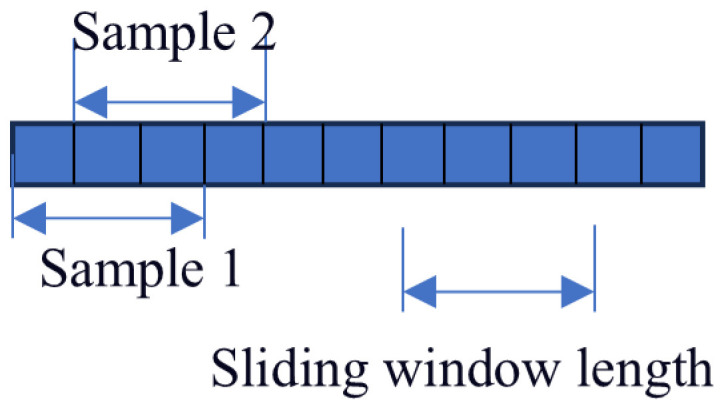
Schematic diagram of sample count in sliding window.

The length of the sliding window significantly affects the accuracy of the performed predictions. A too short window may reduce the complexity of network training. However, it fails to accurately capture potential correlations between multiple time steps. On the other hand, a too long sliding window can better capture the relationship between fault information and time, at the expense of increasing the network training time. Based on the results obtained in [36], the window length and sliding window step size are set to 30 and 1 samples, respectively. A detailed description of this process is shown in Figure 3.

Bearing 1-1 from the PHM2012 dataset is considered as an example. This dataset contains 2803 samples, each one consisting of 2560 data points. A sliding window is applied to form a new sample from the combination of every 30 samples. This yields 2773 new samples, since the sliding window moves by one sample at a time, and therefore the total number of samples decreases by 30 (i.e., 2803 − 30 = 2773). 

The steps for determining bearing failure using the 3σ method combined with the quartile method within a sliding window are as follows:

(1) Calculate the mean μ(t) and standard deviation σ(t) for each window [32]:
  (31) $$\mu (t)=\frac{1}{Z}\sum_{\left(i_{z}-t)-(L+1\right)}^{t}x_{i_{z}}$$
  where *Z* is the length of the sampling window, and $x_{i_{z}}$ denotes the sampled data points.
  (32) $$\sigma (t)=\sqrt{\frac{1}{L-1}\sum_{\left(i_{z}-t)-(L+1\right)}^{t}\;x_{i}{\,}_{z}-\mu {\left(t\right)}^{2})}$$
(2) If a certain point $x_{i}{\,}_{z}$ satisfies condition $\left|x_{i_{z}}-\mu (t)|\geq 3\sigma (t\right)$, according to the 3*σ* principle, the data point is considered anomalous [32].
(3) Sort the *n_h_* data points within the window in ascending order to obtain the ordered set {$x_{1},x_{1},\ldots ,x_{n_{h}}$}. Calculate the first quartile (*f_Q_*_1_) and the third quartile (*f_Q_*_3_) [25]:
  (33) $$f_{Q1}=(n_{h}+1)\times 0.25$$
  (34) $$f_{Q3}=(n_{h}+1)\times 0.75$$
  In the above formula, if *f* is an integer, directly take the value at the corresponding position. If *f* is a decimal, compute the value using linear interpolation [25]:
  *Q* = *x*_⌊*f*⌋_ + (*f* − ⌊*f*⌋) (*x*_⌊*f*⌋+1_ + *x*_⌊*f*⌋_) (35)
  Calculate the interquartile range (I*_QR_*) [25]:
  (36) $$I_{QR}=f_{Q3}-f_{Q1}$$
  If the condition $x_{i_{z}}>f_{Q3}+1.5I_{QR}$ or the condition $x_{i_{z}}<f_{Q3}-1.5I_{QR}$ is satisfied, the data point is considered anomalous.
(4) If data anomalies are detected in either Step (2) or Step (3), the bearing is considered to have failed.

### 3.2. Method for Optimizing BiLSTM Networks Using the Sparrow Search Algorithm (SSA)

In the RUL prediction stage, the SSA is used to adaptively search for the hyperparameters of the model. This aims at ensuring high prediction performance and generalization ability. The Adam algorithm has advantages in the process of updating model parameters, continuously adjusting parameters during model training to approximate local optimal solutions and thus finding a balance between global and local optimality. The Adam optimizer [36] is employed to optimize the weights and biases of the model, which enables efficient model convergence. 

#### 3.2.1. Sparrow Search Algorithm (SSA)

The adopted process is summarized as follows:

(1) The SSA objective function is considered as the prediction error on the test set, the reciprocal of the MSE is used as the fitness value. The MSE is expressed as [36]
  (37) $$MSE=\frac{1}{n_{y}}\sum_{y=1}^{n_{y}}{\left(Y_{t}-\hat{Y_{t}}\right)}^{2}$$
  where *n_y_* represents the number of test samples, and *y* represents the serial number of the data point [38].
  The fitness value is given by [36]
  (38) $$f_{fitness}=\frac{1}{MSE}$$

(2) The position update rule for discoverers in the SSA algorithm is expressed as [36]
  (39) $$G_{a,b}^{d+1}=\left\{\begin{array}{c} G_{a,b}^{d}\cdot \exp(\frac{-a}{\zeta V}),\cup <\partial \\ G_{a,b}^{d}+Z_{0}\cdot A_{0},\cup \geq \partial \end{array}\right.$$
  where *d* represents the current iteration number, *V* represents the total number of iterations, $G_{a,b}^{d+1}$ represents the position information of the *a*th sparrow in the *b*th dimension at the *d*th iteration, ζ represents a random number in the range of 0–1, ∪ represents the warning value in the range of 0–1, ∂ represents the safety value in the range of 0.5–1, Z_0_ represents a random number following normal distribution, and *A*_0_ represents a multi-dimensional row matrix of all ones.

(3) The position update formula for followers is given by [36]
  (40) $$G_{a,b}^{d+1}=\left\{\begin{array}{c} Z_{0}\cdot \exp(\frac{G_{r}^{d}-G_{a,b}^{d}}{n^{2}}),a>\frac{o}{2}\\ G_{e}^{d+1}+\left|G_{a,b}^{d}-G_{e}^{d+1}\right|\cdot H^{+}\cdot A_{0},\mathrm{otherwise} \end{array}\right.$$
  where $G_{e}^{d+1}$ represents the best position occupied by discoverers, $G_{r}^{d}$ represents the global worst position, $H^{+}$ represents a matrix of all ones comprising elements randomly set to 0 or 1, and $H^{+}=H^{T}{\left(HH^{T}\right)}^{-1}$. Note that, for $a>\frac{o}{2}$, half of the following sparrows cannot share the optimal position of the discoverer, and they should search on their own.

Assuming that alerters occupy 10–20% of the population and their initial positions are random, their position update can be expressed as [36]

(41) $$G_{a,b}^{d+1}=\left\{\begin{array}{c} G_{best}^{d}+\tau \left|G_{a,b}^{d}-G_{best}^{d}\right|,f_{fi}>f_{be}\\ G_{e,b}^{d}+\omega \cdot \frac{\left|G_{a,b}^{d}-G_{r}^{d}\right|}{f_{fi}-f_{wo}+\upsilon_{0}},f_{fi}=f_{be} \end{array}\right.$$

where $G_{best}^{d}$ represents the current global optimal position, τ represents a random number of standard normal distribution, ω∈[−1,1], $f_{fi}$, $f_{be}$, and $f_{wo}$, respectively, represent the current alerter sparrow, current global best and worst fitness values, and $\upsilon_{0}$ represents a very small constant used to prevent a null denominator. 

The optimized hyperparameters consist of a number of hidden layer neurons ([16, 512]), initial learning rate ([10^−4^, 1]), batch size ([16, 512]), number of iterations ([50, 3000]), dropout ratio ([0, 0.5]), and number of BiLSTM network layers ([1, 4]) [36,37,38]. 

Assuming the population size of the Sparrow Search Algorithm is *N_SSA_*, the number of iterations is *T_SSA_*, and the problem dimension is *D_w_*, then the time complexity of the SSA algorithm is [36]

(42) $$C_{SSA}=O\left(N_{SSA}\times T_{SSA}\times f(D_{w})+D_{w}\right)$$

where $f(D_{w})$ is the complexity of fitness, $f(D_{w})=O\left(D_{w}^{2}\right)$. 

**Figure 4:**
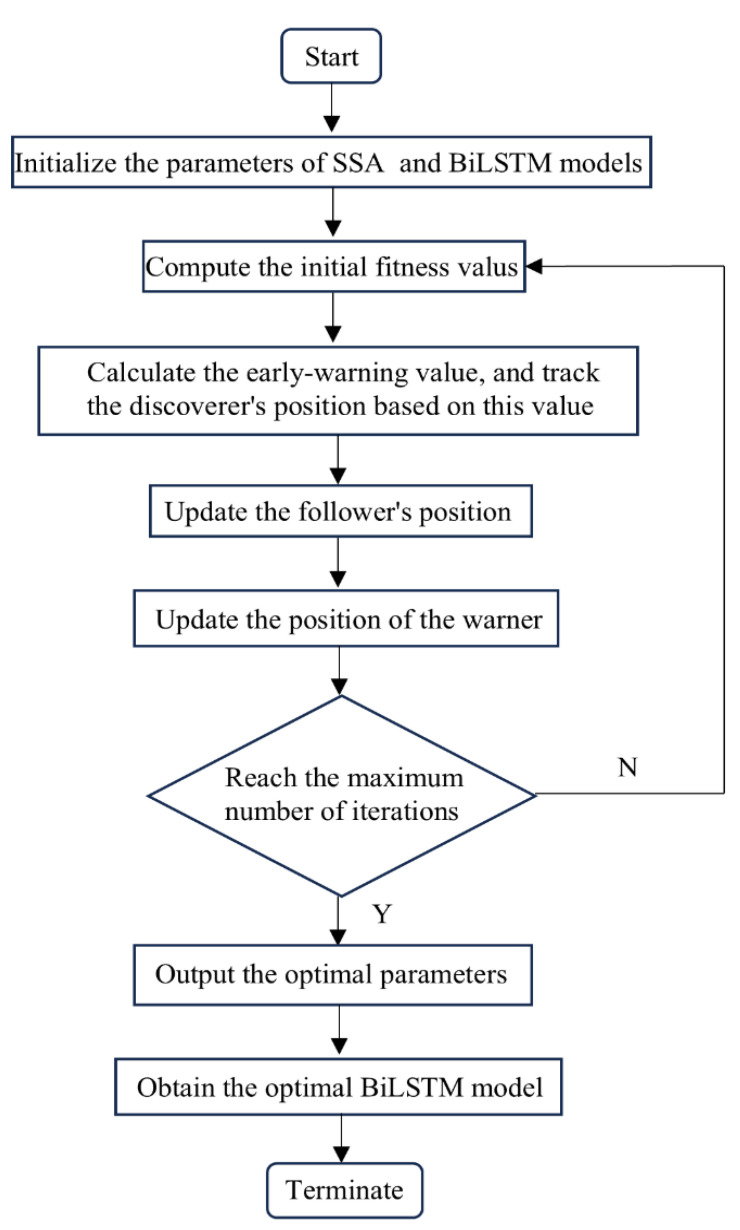
The overall optimization process.

The overall optimization process is illustrated in Figure 4. 

#### 3.2.2. BiLSTM Network Algorithm

**Figure 5:**
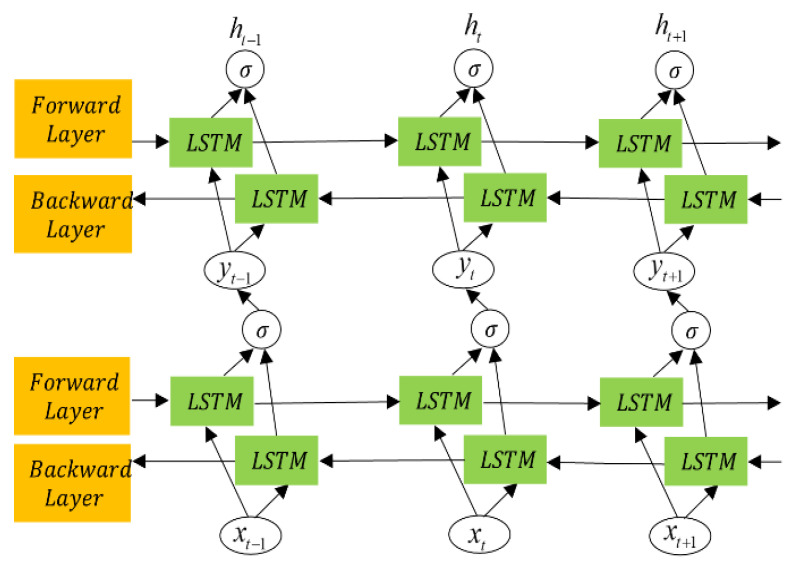
Memory cell of LSTM network.

As shown in Figure 5, it is the optimized BiLSTM network, which two BiLSTM layers stacked to form a dual BiLSTM network, where the output of the first BiLSTM layer serves as the input for the second one. This structure combines BiLSTM networks with a multi-layer stacked architecture. Compared with traditional long LSTM networks and BiLSTM networks, the dual-layer BiLSTM provides unique advantages in bearing life prediction: higher feature extraction capacity, adaptability to complex data, higher prediction accuracy. 

The input to the network *Y* = [*Y*_1_, *Y*_2_,.…, *Y_n_*] is transformed, through iterative mapping, into the output *h* = [*h*_1_, *h*_2_,.…, *h_n_*] over time steps *t* = [1, 2,.…, *t* − 1,.…, *T*]. The corresponding gates in the LSTM unit are expressed as [38]

(43) $$i_{t}=\sigma (\omega_{ix}x_{t}+\omega_{ih}h_{t-1}+b_{i})$$

(44) $$f_{t}=\sigma (\omega_{fx}x_{t}+\omega_{fh}h_{t-1}+b_{f})$$

(45) $$g_{t}=\varphi (\omega_{gx}x_{t}+\omega_{gh}h_{t-1}+b_{g})$$

(46) $$o_{t}=\sigma (\omega_{ox}x_{t}+\omega_{oh}h_{t-1}+b_{o})$$

(47) $$c_{t}=\sigma (g_{t}⊗i_{t}+\omega_{ih}⊗c_{t-1})$$

(48) $$h_{t}=\varphi (c_{t})⊗o_{t}$$

where $\omega_{ix}$, $\omega_{fx}$, $\omega_{gx}$, and $\omega_{ox}$ represent the weight matrices between the input layer and corresponding gates at time *t*; $\omega_{ih}$, $\omega_{fh}$, $\omega_{gh}$, and $\omega_{oh}$ represent the weight matrices of the hidden layer between time values *t* and *t* – 1; $b_{i}$, $b_{f}$, $b_{g}$, and $b_{o}$, respectively, represent the bias values of the input, forget, control, and output gates; $h_{t-1}$ and $c_{t-1}$, respectively, represent the hidden and cell states of the previous time value *t* – 1; $i_{t}$, $f_{t}$, $g_{t}$, and $o_{t}$, respectively, represent the output values of the input, forget, control, and output gates; $c_{t}$ and $h_{t}$, respectively, represent the current cell state and hidden state at time *t*; φ and σ, respectively, represent the tanh and sigmoid activation functions; and ⊗ represents the point-wise multiplication operator [38]. 

The dual-layer BiLSTM generates hidden sequences in forward and backward directions. The forward and backward hidden sequences are denoted by $\vec{h}=[\vec{h},\vec{h},\ldots \vec{h}]$ and $\overset{\leftarrow }{h}=[\overset{\leftarrow }{h},\overset{\leftarrow }{h},\ldots \overset{\leftarrow }{h}]$, respectively. The final output is determined by concatenating the forward and backward outputs. The encoded vectors generated by the two hidden layers are expressed as [38]

(49) $$y_{t}=\sigma (\omega_{y\vec{h}}{\vec{h}}_{t}+\omega_{y\overset{\leftarrow }{h}}{\overset{\leftarrow }{h}}_{t}+b_{y})$$

(50) $${\vec{h}}_{t}=\sigma (\omega_{\vec{h}x}x_{t}+\omega_{\vec{h}\vec{h}}{\vec{h}}_{t-1}+b_{\vec{h}})$$

(51) $${\overset{\leftarrow }{h}}_{t}=\sigma (\omega_{\overset{\leftarrow }{h}x}x_{t}+\omega_{\overset{\leftarrow }{h}\overset{\leftarrow }{h}}h_{t+1}+b_{\overset{\leftarrow }{h}})$$

(52) $$h_{t}=\omega_{\vec{h}\vec{h}}{\vec{h}}_{t}+\omega_{\overset{\leftarrow }{h}\overset{\leftarrow }{h}}{\overset{\leftarrow }{h}}_{t}+b_{h}$$

where $y_{t}=[\vec{h},\overset{\leftarrow }{h}]$ represents the output of the network expressed in the first hidden layer as *y_t_* = [*y*_1_, *y*_2_,.…, *y_t_*,.…, *yn*] [38]. 

**Figure 6:**
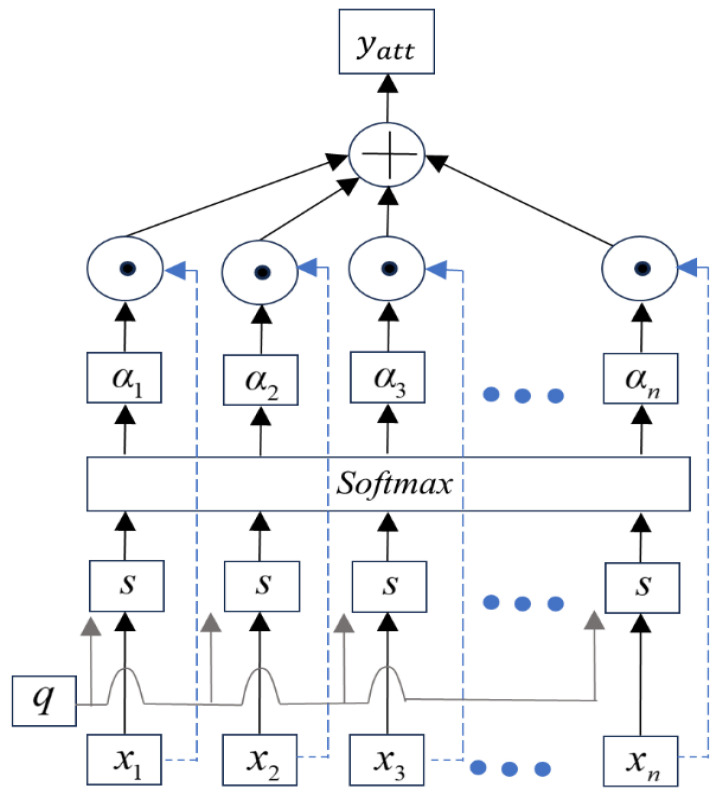
Structure of the attention mechanism network.

When training data using the BiLSTM model, the attention mechanism [38] can directly model the relationships between any two time steps, allowing to better capture long-range dependencies. This helps the model extract the most critical information from these complex signals for predicting the RUL of bearings [1,2,3,38]. Figure 6 illustrates the network structure, where the output of the attention layer is given by [15]

(53) $$\alpha_{i}=p(m=i|n,q)=\frac{\exp(s(x_{i},q))}{\sum_{j=1}^{n}\exp(s(x_{i},q))}$$

where $s(x_{i},q)$ represents the scoring function, and $\alpha_{i}$ represents the attention coefficient [38]. 

$s(x_{i},q)$ is given by [15]

(54) $$s(x_{i},q)=q^{T}x_{i}$$

The output of the attention mechanism is given by [15]

(55) $$y_{att}=\sum_{i=1}^{n}\alpha_{n}x_{n}$$

For the output values of the BiLSTM layer, the output of the attention mechanism is given by [15]

(56) α=Softmax(s(H,q))

(57) $$y_{att}=\tanh(H\alpha^{T})$$

The final output *y_att_* represents the high-level abstract information of the input, which is used to evaluate the bearing degradation function of time [17,38]. 

The weights and biases of the network are optimized using the Adam optimizer. The Adam optimization is expressed as [36]

(58) $$\left\{\begin{array}{c} u_{t}=\beta_{1}u_{t-1}+(1-\beta_{1})g_{t}\\ v_{t}=\beta_{2}v_{t-1}+(1-\beta_{2})g^{2}{\,}_{t}\\ \hat{u_{t}}=\frac{u_{t}}{1-\beta^{t}{\,}_{1}}\\ \hat{v_{t}}=\frac{v_{t}}{1-\beta^{t}{\,}_{2}}\\ w_{t}=w_{t-1}+\frac{a}{\sqrt{\hat{v_{t}}+\varepsilon }}\hat{u_{t}}\\ g=\frac{1}{u}\nabla_{w_{t}}\sum_{i}L(f(x^{\left(i\right)};w_{t}),y^{\left(i\right)}) \end{array}\right.$$

where $u_{t}$ and $v_{t}$, respectively, represent the estimates of the first-order and second-order moments of the gradient; ${\hat{u}}_{t}$ and ${\hat{v}}_{t}$ are, respectively, the corrected values; $L(f(x^{\left(i\right)};w_{t}),y^{\left(i\right)})$ represents the training sample; $x^{\left(i\right)}$, *w_t_*, and $y^{\left(i\right)}$ are, respectively, the input, weight, and output values; *g* represents the gradient; *a_0_* represents the learning rate; $\beta_{1}$ and $\beta_{2}$, respectively, represent the gradient and squared gradient decay factors; and ε is a very small constant used for numerical stability (usually set to ε = $10^{-8}$ [36]). 

### 3.3. RUL Prediction Model

**Figure 7:**
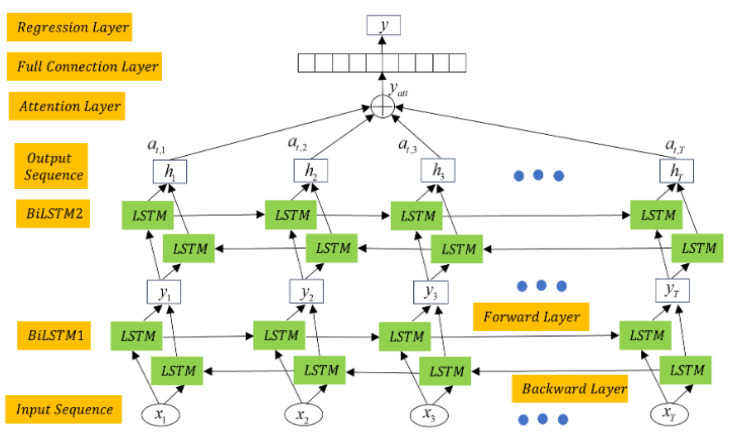
The network architecture combining BiLSTM with the attention mechanism.

The RUL prediction of bearings mainly includes the training and testing stages. The training dataset ${\left\{X_{t},Y_{t}\right\}}_{t=1}^{T}$ serves as the input to the network [38], where $X_{t}\in {ℜ}^{N\times 1}$ contains *N* sensitive features at time *t*, and *Yt* represents the actual labels related to the degradation of the bearing at this time. By stacking two BiLSTM network layers, the degradation information of the bearing is propagated in both forward and backward directions. As shown in Figure 7, the structure combines two BiLSTM network layers with an attention mechanism. The output of BiLSTM1 is the input of BiLSTM2. The final hidden states are multiplied by the (*a_t_*_,1_, *a_t_*_,2_, *a_t_*_,3_, …, *a_t_*_,*T*_) attention weights and summed to generate the final output of the network, *y_att_* [38]. The RUL prediction value is then determined through a regression layer. In the latter, the model is optimized using the mean squared error (MSE) [36]:

(59) $$LL=\frac{1}{T}{\sum_{t=1}^{T}\left\|Y_{t}-{\hat{Y}}_{t}\right\|}_{2}^{2}$$

where *Y_t_* represents the ground truth labels of the input data, and ${\hat{Y}}_{t}$ represents the values predicted by the BiLSTM. In the testing stage, the fused features are directly input into the trained model, yielding the RUL prediction values [38]. 

## 4. Experimental Validation and Result Analysis

### 4.1. Introduction to the Experimental Platform

**Figure 8:**
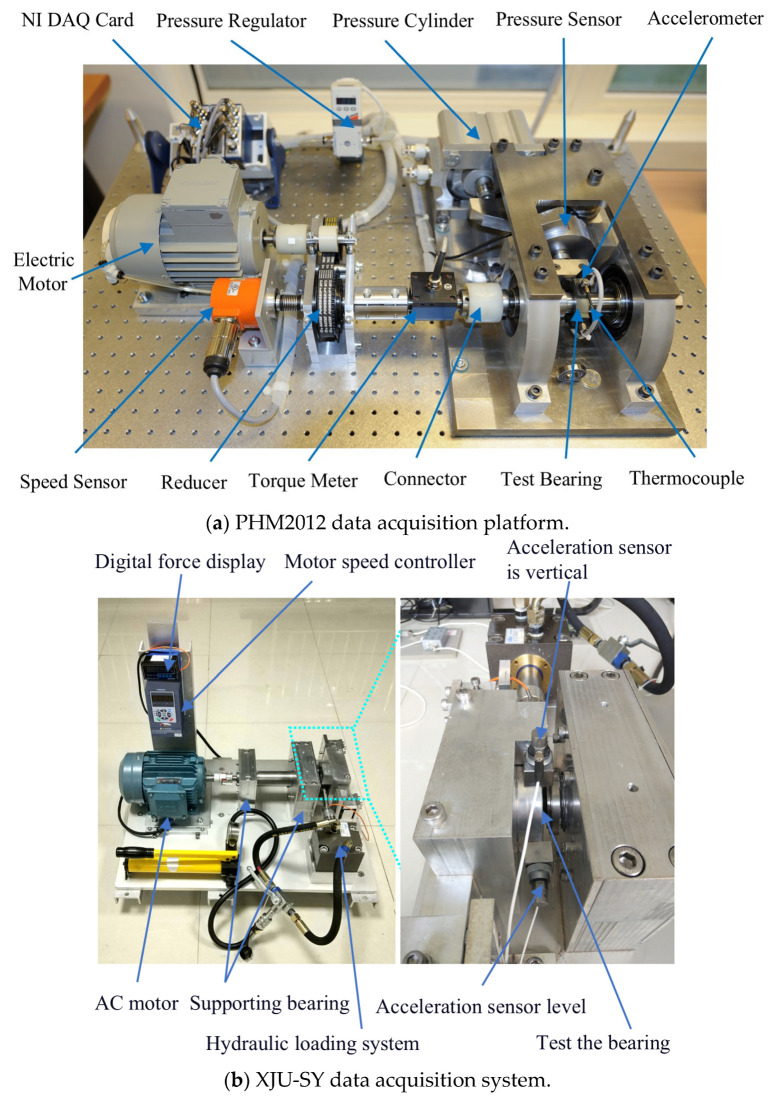
Data acquisition platform.

The IEEE PHM2012 [36] and XJU-SY [36] datasets were then used. The experimental platforms are shown in Figure 8a,b, and the training and test sets are shown in Table 2. PHM2012 has a sampling frequency of 25.6 KHz [38], yielding 2560 data points every 10 s. XJU-SY has a sampling frequency of 25.6 KHZ [38], yielding 32,786 data points every 1 min, with a sampling duration of 1.28 s. The two datasets use horizontal vibration signals for experiments [12]. 

### 4.2. Analysis of Data Preprocessing Results

#### 4.2.1. Analysis of Denoising Results

**Figure 9:**
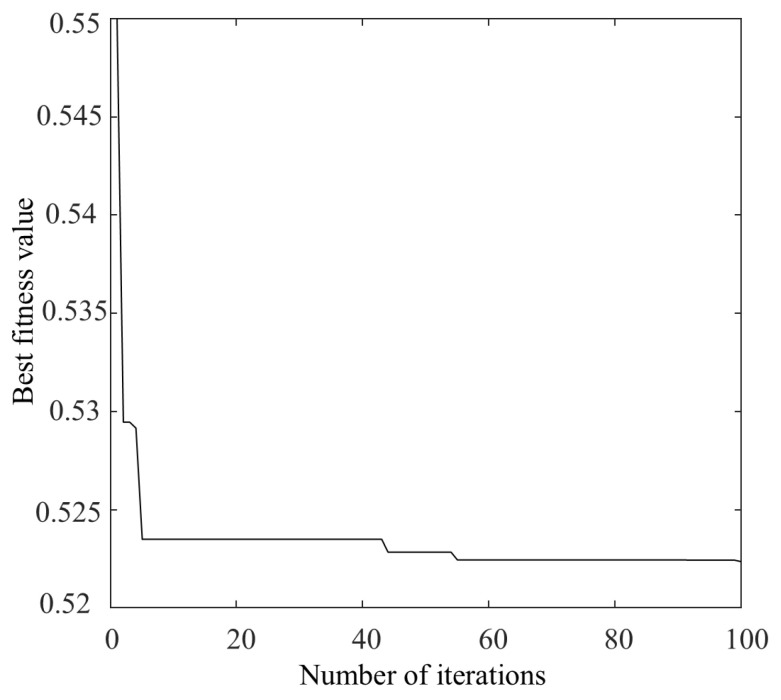
Best fitness value curve.

Bearing 1-3 of the PHM2012 was considered as an example, on which denoising was performed. When optimizing the parameters of VMD using the NGO algorithm, 30 different initial populations were randomly generated, with each population consisting of 20 individuals. The initial ranges for α and k were set to [1, 20] and [500, 2500], respectively. The algorithm was run for 100 iterations in each case. For each run, the mean and standard deviation of the best fitness values, as well as the mean and standard deviation of the optimal parameters [α, k], were recorded across 30 populations during independent runs. If the standard deviation is small, it indicates that the algorithm has good stability and global convergence performance. The optimal values of [α, k] were ultimately obtained as [8, 1879], and the optimal fitness curve is shown in Figure 9. 

**Figure 10:**
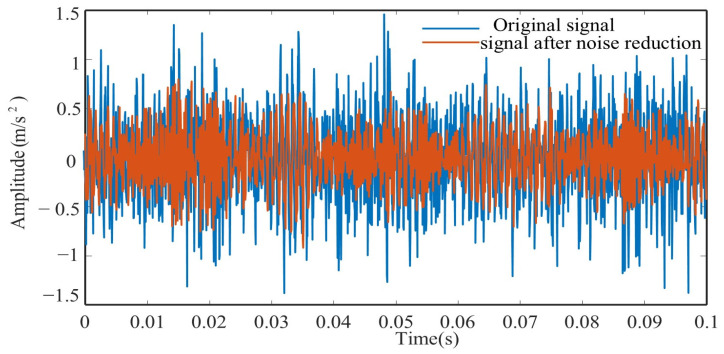
Denoising result.

Figure 10 shows a comparison between vibration signals before and after denoising. Table 3 presents the evaluation indicators calculated on the results obtained by the proposed denoising method and soft-threshold denoising method. The proposed method exhibits higher values of NM and SNR compared to the benchmark methods, demonstrating its superior denoising capability. 

#### 4.2.2. Analysis of Feature Selection Results

**Figure 11:**
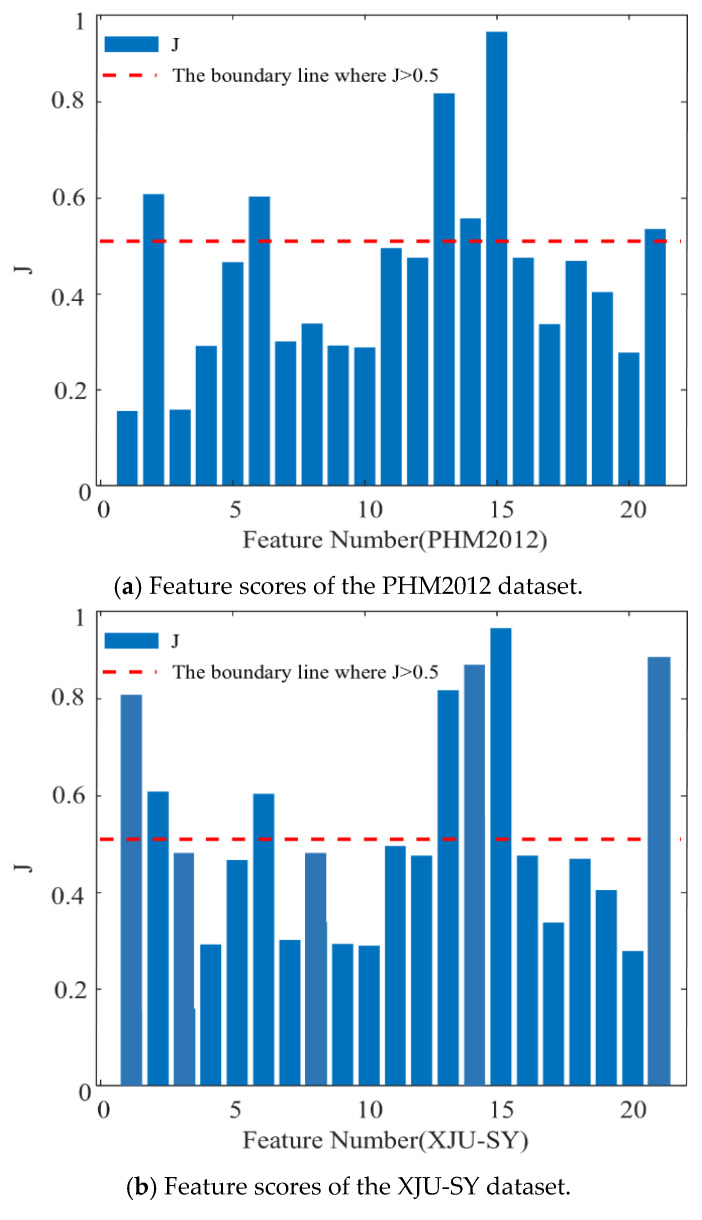
Feature scores.

Taking bearing 1-3 from the PHM2012 dataset and bearing 1-3 from the XJU-SY dataset as examples, the score values of each feature are, respectively, illustrated in Figure 11a,b, the red curve represents the score value of 0.5. It can be seen from Figure 11a that the p_2_, p_6_, p_13_, p_14_, p_15_, and p_21_ extracted features of PHM2012 have score values greater than 0.5. Therefore, these features are considered optimal features. It can be observed from Figure 11b that the p_1_, p_2_, p_6_, p_13_, p_14_, p_15_, and p_21_ extracted features of XJU-SY have score values greater than 0.5, and thus they are considered optimal features. These features contain rich degradation information of bearings, allowing them to contribute effectively to the performed RUL prediction. 

To verify that the selected features are the optimal ones for RUL prediction, ablation experiments were conducted using the following methods: (1) Input the selected feature (represented by *p*) and all features (represented by *p_all_*) into the RUL prediction model to obtain the prediction results. (2) Remove one of the selected features at a time and input the remaining features (represented by *p* − *p_io_*, where *io* stands for feature number) into the RUL prediction model to obtain the prediction results. The Root Mean Square Error (RMSE) was used as the evaluation criterion for both steps.

(60) $$RMSE=\sqrt{\frac{1}{n_{y}}{\sum_{y=1}^{n_{y}}\left|Y_{t}-\hat{Y_{t}}\right|}^{2}}$$

Due to the large amount of data in the experimental results, the ablation experiment results for Bearings 1-3 are presented for each dataset. As shown in Table 4, which presents the results after conducting the ablation experiments, when performing RUL prediction using the selected features versus all features, there is little difference in their RMSE values. This indicates that both have a similar impact on the RUL prediction results. To reduce data redundancy, it is sufficient to use the selected features for RUL prediction. When any one feature is removed from the selected features, the RMSE fluctuates significantly, and the RMSE value calculated using the selected features is smaller. A smaller RMSE value indicates more accurate RUL prediction results. Therefore, based on the comprehensive experimental results above, it can be concluded that using the selected features for RUL prediction yields good predictive performance. 

### 4.3. HI and Failure Threshold Results

**Figure 12:**
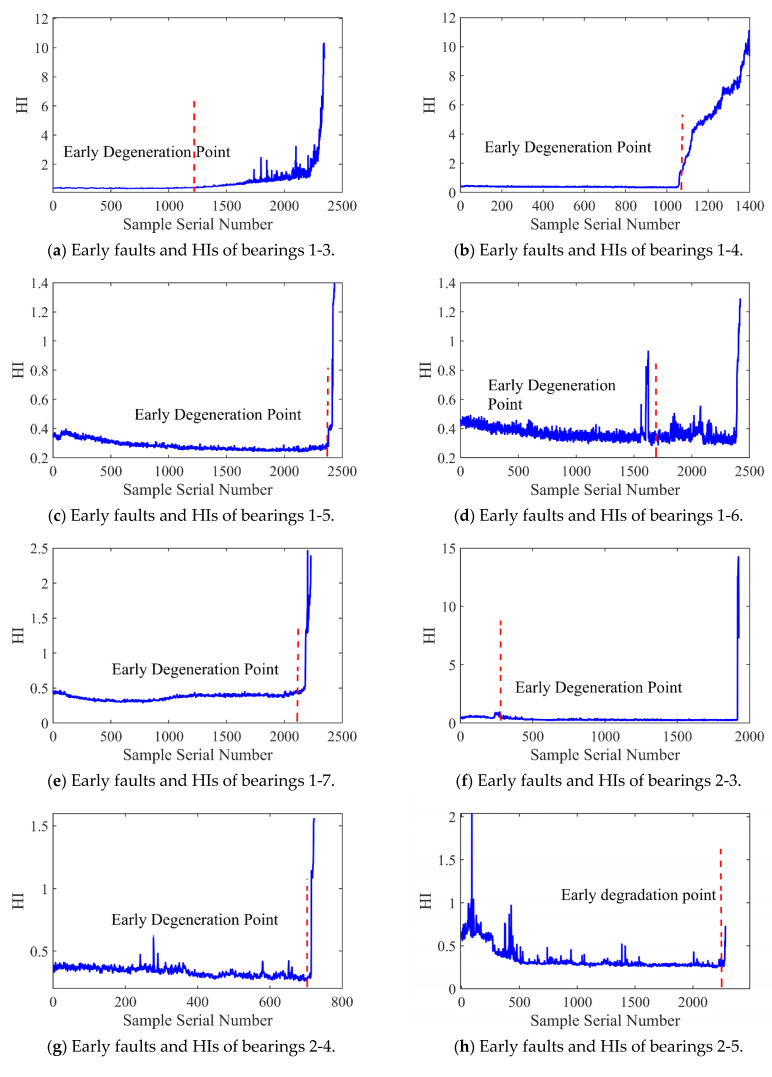

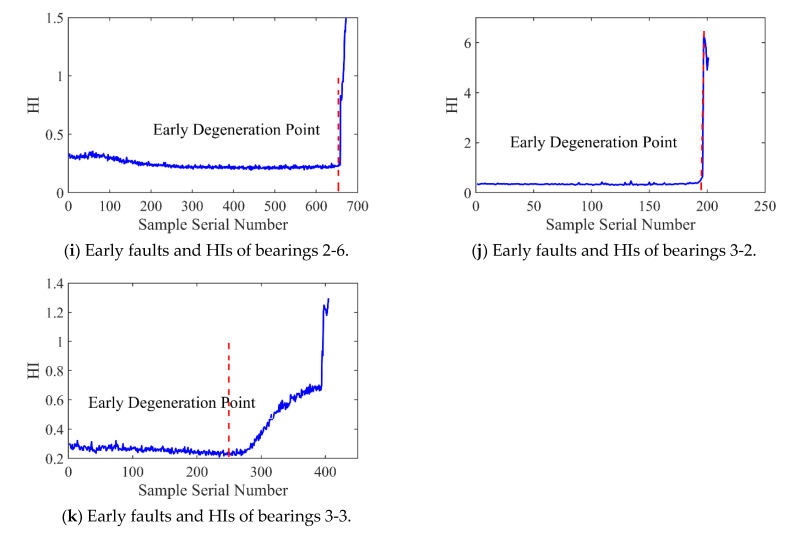
Early faults and HIs for PHM2012.

**Figure 13:**
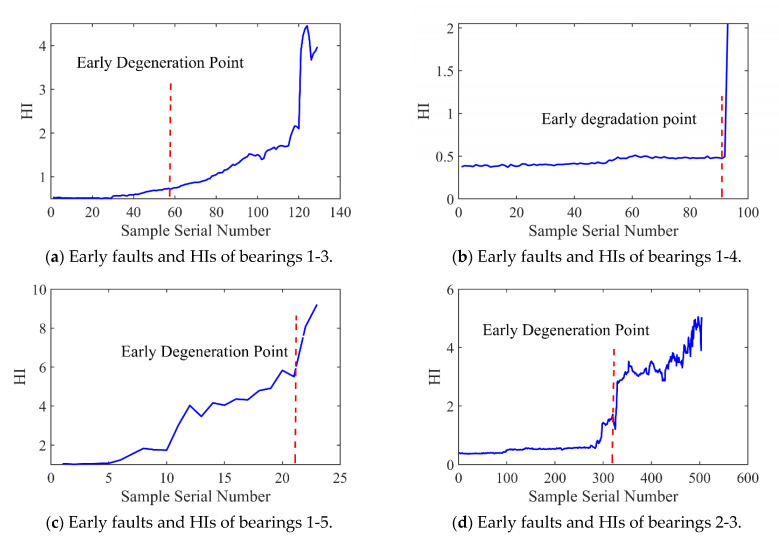

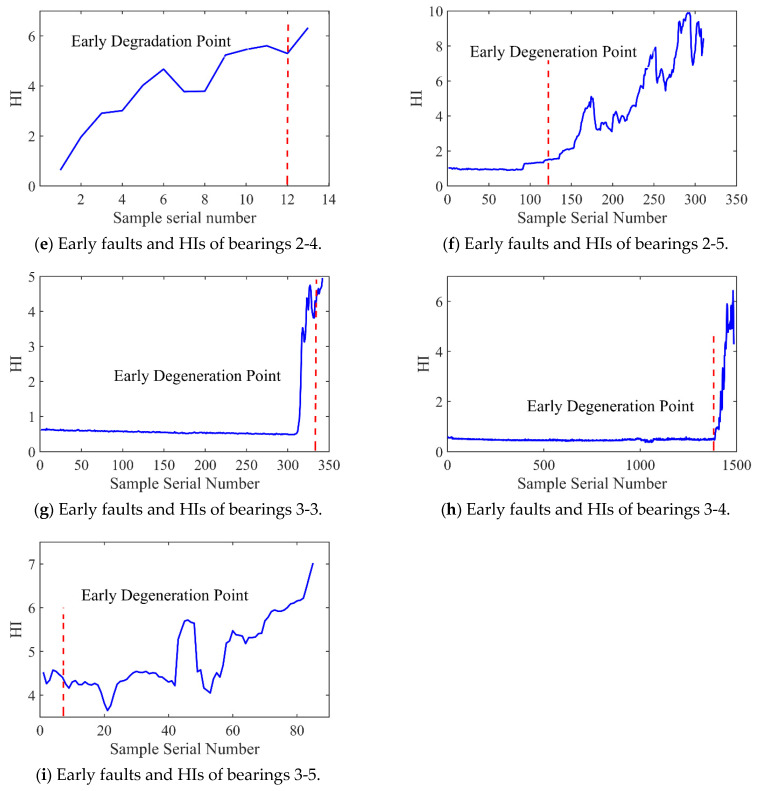
Early faults and HIs for XJU-SY.

Figure 12 and Figure 13 present the HI and early degradation points of the test sets for the PHM2012 and XJU-SY datasets, respectively, the red curve represents the failure point of the bearing. Table 5 shows the bearing early degradation point detection results obtained using the dynamic threshold and fixed threshold methods. 

The fixed threshold method failed to detect the early degradation points of bearings 2-4 and 2-5 in the PHM dataset, as well as those of bearings 1-4 and 1-5 in the XJU-SY dataset. In contrast, the proposed method could detect early degradation points across all datasets. This is due to the fact that the dynamic threshold detection method can adaptively adjust to different working conditions and operational degradation stages of the bearing, and it can perform a real-time adjustment of the degradation threshold of the bearing to adapt to the whole process of its life, enabling early degradation detection and eliminating the risk of missed detections. 

### 4.4. Analysis of RUL Prediction Results

Table 6 shows the detailed network parameters of the proposed RUL prediction model and those of CNN-BiLSTM and stacked BiGRU (stacking two layers of BiGRU networks) used for comparison. 

Table 7 presents the time complexity of SSA-optimized BiLSTM. Both the SSA search time and inference latency are average times, measured in seconds (s). 

**Figure 14:**
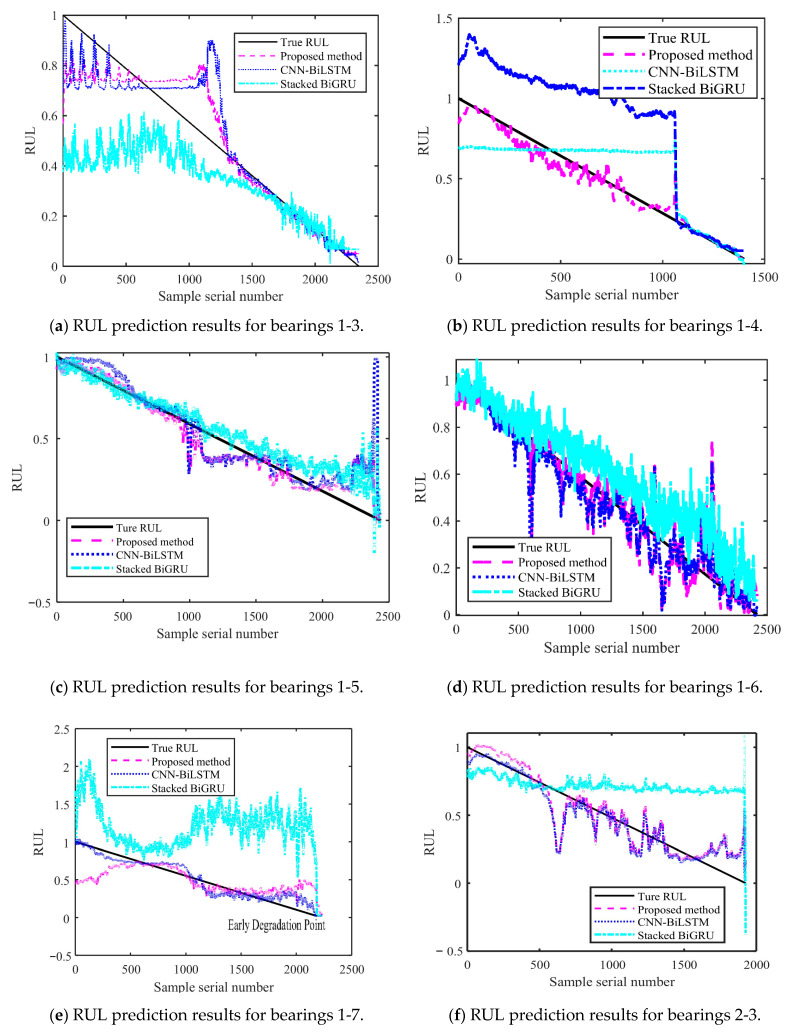

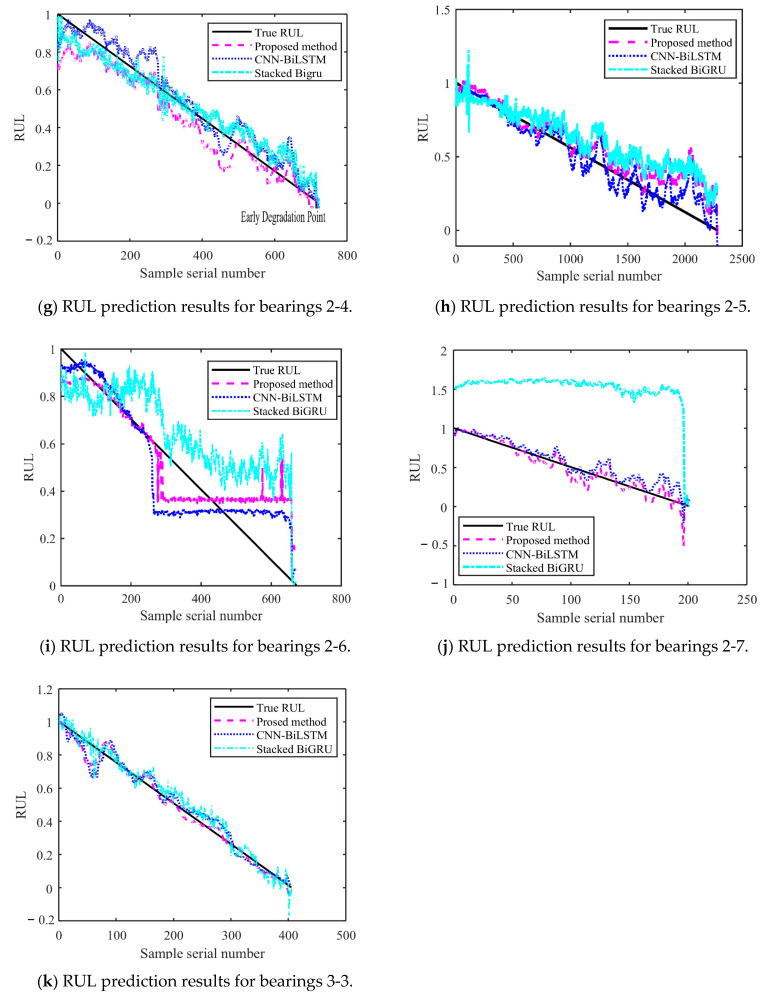
RUL prediction results for PHM2012.

**Figure 15:**
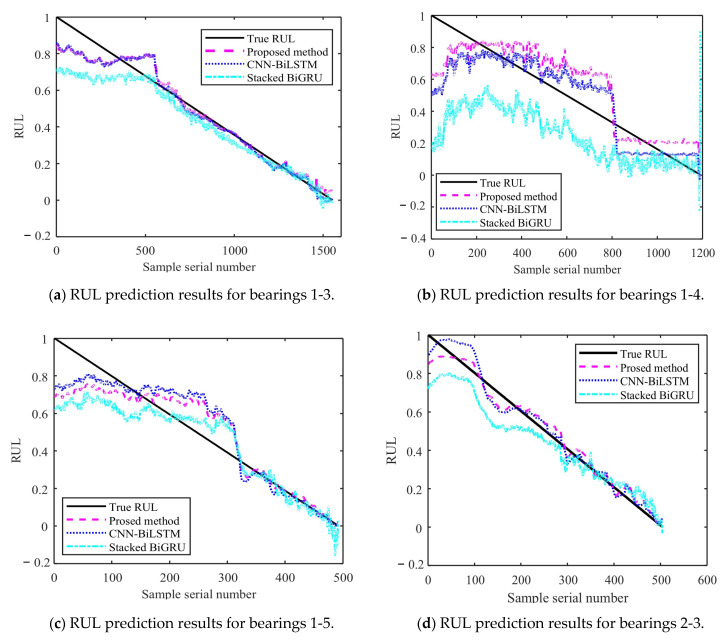

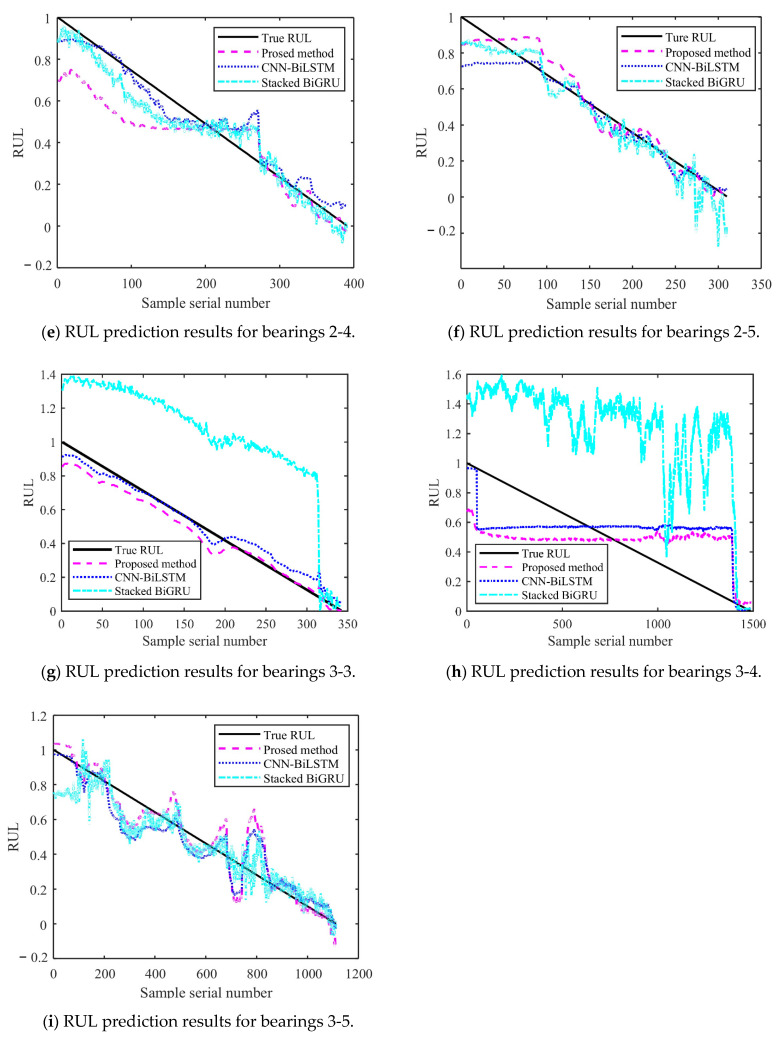
RUL prediction results for XJU-SY.

Figure 14 and Figure 15 show the results of the test set obtained by testing on PHM2012 and XJU-SY, respectively. It can be seen that the RUL prediction curves exhibit a monotonically decreasing trend. The analysis presented in Section 4.3 demonstrates that, during the operation of bearings, their performance is gradually degraded because of many factors, such as wear, fatigue, and corrosion. Over time, the degradation effects accumulate, leading to a further decrease in bearing performance. Therefore, when the bearing service time increases, its remaining life time is gradually shortened. It can also be seen from the RUL prediction curves that the three methods can predict the RUL of bearings. However, in general, the proposed method exhibits a smaller curve fluctuation range compared to those of the other methods, which demonstrates that it has higher stability. 

The Root Mean Square Error (RMSE) emphasizes the impact of large errors and facilitates horizontal comparisons with other models. The Mean Absolute Error (MAE) provides an intuitive measure of the average prediction error and can more robustly evaluate the performance of the model. The Coefficient of Determination (R^2^) measures the degree of fit of the model to the data, with a value closer to 1 indicating better fit. It is suitable for scenarios where it is necessary to compare the fitting effects of different models. The formula for RMSE has already been provided in Section 4.2.2. The formulas for MAE and R^2^ are as follows, respectively [36]:

(61) $$MAE=\frac{1}{n_{y}}\sum_{y=1}^{n_{y}}\left|Y_{t}-\hat{Y_{t}}\right|$$

(62) $$R^{2}=1-\frac{\sum_{y=1}^{n_{y}}{\left(Y_{t}-\hat{Y_{t}}\right)}^{2}}{\sum_{y=1}^{n_{y}}{\left(Y_{t}-\overset{-}{Y_{t}}\right)}^{2}}$$

The obtained MAE, RMSE, and R^2^ values are presented in Table 8. It can be seen that the MAE and RMSE of the BiLSTM model are smaller than those of the other two methods, demonstrating higher performance. The R^2^ value of the BiLSTM model is greater than that of the other methods. This shows that the fitting degree of the proposed network model is higher than those of the other methods. By predicting the bearing RUL, a preventive maintenance of machines can be performed, providing time for staff to establishing repair strategies and reducing the economic losses caused by failures. When inputting two different datasets into the proposed RUL prediction model, it demonstrates high prediction ability. This shows that it has high adaptability and generalization ability, yielding prediction results that are more stable than those obtained by other methods. 

## 5. Conclusions

The NGO method was used to optimize the VMD algorithm (NVMD). The Pearson correlation coefficient was adopted to denoise the original vibration signals of bearings. The obtained results were compared with those of the soft-threshold denoising method, and optimal features were selected based on monotonicity, trendability, predictability, and robustness indicators. The KPCA method was used to fuse features in order to determine the HI of the bearing. A dynamic threshold method was proposed to detect the early degradation point of bearings. The obtained results were compared with those of the fixed threshold method. The BiLSTM model was combined with the attention mechanism for RUL prediction. The SSA was used to adaptively find the network parameters, allowing prediction of the bearing RUL. The obtained results were compared with those of the CNN-BiLSTM and stacked BiGRU methods. The main conclusions are summarized as follows:

(1) The proposed method exhibits high denoising capability. The denoising capability significantly affects the quality of the results of bearing RUL prediction. After filtering using the NVMD method, the bearing signal becomes purer, which improves its quality for the subsequent bearing signal feature extraction, early degradation detection, and life prediction.
(2) Different bearing features encapsulate degradation information from various aspects. During feature selection, it is necessary to comprehensively consider multiple features. Based on the ablation experiment results across different features, the features extracted using the proposed method contain richer degradation information. By fusing only these sensitive features, accurate prediction can be performed for RUL prediction.
(3) Early degradation time detection is a crucial parameter in the evaluation of the bearing performance. Since the characteristics of bearing vibration signals change in real time, the degradation threshold of the signal also varies over time. Using a dynamic threshold setting method to determine the degradation threshold of bearing vibration signals, real-time updates of the mean and standard deviation can be performed. This allows us to update the failure threshold in real time. Compared with the fixed threshold method, the dynamic threshold method can detect the bearing degradation time earlier, enabling the preventive maintenance of bearings.
(4) A comparison was conducted among the RUL, CNN-BiLSTM, and stacked BiGRU prediction performances. The obtained results showed that the proposed RUL prediction method demonstrates higher performance and stronger fitting ability. This demonstrates that it has higher effectiveness in predicting RUL. The proposed model was then applied on the PHM2012 and XJU-SY datasets, demonstrating its high generalization ability and performance. The use of the proposed method for RUL prediction allows for timely detection of potential bearing faults. This provides sufficient time for performing timely bearing repair, guiding staff in formulating complete maintenance measures.

## Figures and Tables

**Table 1:** Feature expressions.

Serial Number	Name	Feature Expression
1	Mean	$p_{1}=\frac{\sum_{i=1}^{N}x_{i}}{N}$
2	Standard Deviation	$p_{2}=\sqrt{\frac{\sum_{i=1}^{N}{\left(x_{i}-p_{1}\right)}^{2}}{N}}$
3	Variance	$p_{3}=\frac{\sum_{i=1}^{N}{\left(x_{i}-p_{1}\right)}^{2}}{N-1}$
4	Skewness	$p_{4}=\frac{\sum_{i=1}^{N}x_{i}^{3}}{N}$
5	Mean Square Root Value	$p_{5}=\sqrt{{\left(\frac{\sum_{i=1}^{N}x_{i}^{2}}{N}\right)}^{2}}$
6	Root Mean Square Value	$p_{6}=\sqrt{\frac{\sum_{i=1}^{N}x_{i}^{2}}{N}}$
7	Kurtosis	$p_{7}=\frac{\sum_{i=1}^{N}x_{i}^{4}}{N}$
8	Waveform	$p_{8}=\frac{p_{5}}{\left|p_{1}\right|}$
9	Kurtosis	$p_{9}=\frac{p_{7}}{\left|p_{6}\right|}$
10	Skewness	$p_{10}=\frac{N}{\left(N-1)(N-2\right)}\sum_{i=1}^{N}(\frac{x_{i}-p_{1}}{p_{2}}{)}^{3}$
11	Peak	$p_{11}=\max(x(t_{i}))$
12	Mean Amplitude in Frequency Domain	$p_{12}=\frac{\sum_{k=1}^{N}s_{k}}{N}$
13	Root Mean Square Frequency	$p_{13}=\frac{\sum_{k=1}^{N}{\left(f_{k}\right)}^{2}s(k)}{\sum_{k=1}^{K}s(k)}$

where *x_i_* is the time-domain signal sequence such that *i* = 1, 2·…, *N* is the number of sample points, *s*(*k*) is the spectrum of signal *x*(*n*), *k* = 1, 2, …, *k* is the number of spectral lines, and *f_k_* is the frequency of the *k*th spectral line [38].

**Table 2:** Bearing dataset [38].

Data	Serial Number	Condition	Training Set	Test Set
PHM2012	Condition 1	4 KN, 1800 rpm	Bearing 1-1 Bearing 1-2	Bearing 1-3, Bearing 1-4 Bearing 1-5, Bearing 1-6 Bearing 1-7
	Condition 2	4.2 KN, 1650 rpm	Bearing 2-1 Bearing 2-2	Bearing 2-3, Bearing 2-4 Bearing 2-5, Bearing 2-6 Bearing 2-7
	Condition 3	5 KN, 1500 rpm	Bearing 3-1 Bearing 3-2	Bearing 3-3
XJU-SY	Condition 1	12 KN, 2100 rpm	Bearing 1-1 Bearing 1-2	Bearing 1-3, Bearing 1-4 Bearing 1-5
	Condition 2	11 KN, 2250 rpm	Bearing 2-1 Bearing 2-2	Bearing 2-3, Bearing 2-4 Bearing 2-5
	Condition 3	10 KN, 2400 rpm	Bearing 3-1 Bearing 3-2	Bearing 3-3, Bearing 3-4 Bearing 3-5

**Table 3:** The result of signal denoising.

	Proposed Method	Soft Thresholding
NM	2.5	1.9
SNR	25	4

**Table 4:** Evaluation results of ablation experiments.

PHM2012		XJU-SY	
Feature RMSE		Feature RMSE	
*p_all_*	0.1035	*p_all_*	0.0642
*p*	0.1033	*p*	0.0630
*p-p*_2_	0.1101	*p-p*_1_	0.0710
*p-p*_6_	0.1042	*p-p*_2_	0.0644
*p-p*_13_	0.1029	*p-p*_6_	0.0649
*p-p*_14_	0.1034	*p-p*_13_	0.0890
*p-p*_15_	0.1039	*p-p*_14_	0.0798
*p-p*_21_	0.1149	*p-p*_15_	0.0644
*p-p*_2_ *-p*_6_	0.1110	*p-p*_21_	0.0654
*p-p*_2_ *-p*_13_	0.1101	*p-p*_1_ *-p*_2_	0.0690
*p-p*_2_ *-p*_14_	0.1102	*p-p*_1_ *-p*_6_	0.0900
*p-p*_2_ *-p*_15_	0.1046	*p-p*_1_ *-p*_13_	0.0789
*p-p*_2_ *-p*_21_	0.1051	*p-p*_1_ *-p*_14_	0.0840
*p-p*_6_ *-p*_13_	0.1046	*p-p*_1_ *-p*_15_	0.0910
*p-p*_6_ *-p*_14_	0.1088	*p-p*_1_ *-p*_21_	0.1002
*p-p*_6_ *-p*_15_	0.1056	*p-p*_2_ *-p*_6_	0.0900
*p-p*_6_ *-p*_21_	0.1062	*p-p*_2_ *-p*_13_	0.0639
*p-p*_13_ *-p*_14_	0.1038	*p-p*_2_ *-p*_14_	0.0832
*p-p*_13_ *-p*_15_	0.1043	*p-p*_2_ *-p*_15_	0.0754
*p-p*_13_ *-p*_21_	0.1066	*p-p*_2_ *-p*_21_	0.0876
*p-p*_14_ *-p*_15_	0.1047	*p-p*_6_ *-p*_13_	0.0655
*p-p*_14_ *-p*_21_	0.1037	*p-p*_6_ *-p*_14_	0.0779
*p-p*_15_ *-p*_21_	0.1114	*p-p*_6_ *-p*_15_	0.0666
*p-p*_2_ *-p*_6_ *-p*_13_	0.1117	*p-p*_6_ *-p*_21_	0.0977
*p-p*_2_ *-p*_6_ *-p*_14_	0.1108	*p-p*_13_ *-p*_14_	0.0962
*p-p*_2_ *-p*_6_ *-p*_15_	0.1109	*p-p*_13_ *-p*_15_	0.0988
*p-p*_2_ *-p*_6_ *-p*_21_	0.1121	*p-p*_13_ *-p*_21_	0.0879
*p-p*_2_ *-p*_13_ *-p*_14_	0.1089	*p-p*_14_ *-p*_15_	0.8567
*p-p*_2_ *-p*_13_ *-p*_15_	0.1077	*p-p*_14_ *-p*_21_	0.9947
*p-p*_2_ *-p*_13_ *-p*_21_	0.1064	*p-p*_15_ *-p*_21_	0.1087
*p-p*_6_ *-p*_13_ *-p*_14_	0.1055	*p-p*_1_ *-p*_2_ *-p*_6_	0.0756
*p-p*_6_ *-p*_14_ *-p*_15_	0.1082	*p-p*_1_ *-p*_2_ *-p*_13_	0.0821
*p-p*_6_ *-p*_15_ *-p*_21_	0.1074	*p-p*_1_ *-p*_2_ *-p*_14_	0.0900
*p-p*_13_ *-p*_14_ *-p*_15_	0.1063	*p-p*_1_ *-p*_2_ *-p*_15_	0.0899
*p-p*_13_ *-p*_14_ *-p*_21_	0.1043	*p-p*_1_ *-p*_2_ *-p*_21_	0.0689
*p-p*_14_ *-p*_15_ *-p*_21_	0.1058	*p-p*_2_ *-p*_6_ *-p*_13_	0.0890
*p-p*_2_ *-p*_6_ *-p*_13_ *-p*_14_	0.1054	*p-p*_2_ *-p*_6_ *-p*_14_	0.0926
*p-p*_2_ *-p*_6_ *-p*_13_ *-p*_15_	0.1070	*p-p*_2_ *-p*_6_ *-p*_15_	0.0920
*p-p*_2_ *-p*_6_ *-p*_13_ *-p*_21_	0.1044	*p-p*_2_ *-p*_6_ *-p*_21_	0.0670
*p-p*_6_ *-p*_13_ *-p*_14_ *-p*_15_	0.1054	*p-p*_6_ *-p*_13_ *-p*_14_	0.8864
*p-p*_6_ *-p*_13_ *-p*_14_ *-p*_21_	0.1099	*p-p*_6_ *-p*_13_ *-p*_15_	0.1179
*p-p*_13_ *-p*_14_ *-p*_15_ *-p*_21_	0.1210	*p-p*_6_ *-p*_13_ *-p*_21_	0.1123
*p-p*_2_ *-p*_6_ *-p*_13_ *-p*_14_ *-p*_15_	0.2440	*p-p*_13_ *-p*_14_ *-p*_15_	0.1243
*p-p*_2_ *-p*_6_ *-p*_13_ *-p*_14_ *-p*_21_	0.1077	*p-p*_13_ *-p*_14_ *-p*_21_	0.1104
*p-p*_2_ *-p*_6_ *-p*_13_ *-p*_14_ *-p*_15_ *-p*_21_	0.1055	*p-p*_14_ *-p*_15_ *-p*_21_	0.1008
		*p-p*_1_ *-p*_2_ *-p*_6_ *-p*_13_	0.0679
		*p-p*_1_ *-p*_2_ *-p*_6_ *-p*_14_	0.0943
		*p-p*_1_ *-p*_2_ *-p*_6_ *-p*_15_	0.0699
		*p-p*_1_ *-p*_2_ *-p*_6_ *-p*_21_	0.0970
		*p-p*_2_ *-p*_6_ *-p*_13_ *-p*_14_	0.0975
		*p-p*_2_ *-p*_6_ *-p*_13_ *-p*_15_	0.0953
		*p-p*_2_ *-p*_6_ *-p*_13_ *-p*_21_	0.0698
		*p-p*_6_ *-p*_13_ *-p*_14_ *-p*_15_	0.0877
		*p-p*_6_ *-p*_13_ *-p*_14_ *-p*_21_	0.0933
		*p-p*_13_ *-p*_14_ *-p*_15_ *-p*_21_	0.1003
		*p-p*_1_ *-p*_2_ *-p*_6_ *-p*_13_ *-p*_14_	0.0999
		*p-p*_1_ *-p*_2_ *-p*_6_ *-p*_13_ *-p*_15_	0.1110
		*p-p*_1_ *-p*_2_ *-p*_6_ *-p*_13_ *-p*_21_	0.1011
		*p-p*_2_ *-p*_6_ *-p*_13_ *-p*_14_ *-p*_15_	0.0955
		*p-p*_2_ *-p*_6_ *-p*_13_ *-p*_14_ *-p*_21_	0.0987
		*p-p*_6_ *-p*_13_ *-p*_14_ *-p*_15_ *-p*_21_	0.1245
		*p-p*_1_ *-p*_2_ *-p*_6_ *-p*_13_ *-p*_14_ *-p*_15_	0.1008
		*p-p*_1_ *-p*_2_ *-p*_6_ *-p*_13_ *-p*_14_ *-p*_21_	0.0865
		*p-p*_1_ *-p*_2_ *-p*_6_ *-p*_13_ *-p*_14_ *-p*_15_ *-p*_21_	0.0954

**Table 5:** Comparison of different thresholding methods.

Bearing	Serial Number	Proposed Method	Fixed 3σ
PHM2012	1-3	1330	1414
	1-4	1072	11,085
	1-5	2423	2428
	1-6	1622	1632
	1-7	2191	2209
	2-3	267	271
	2-4	744	Not Detected
	2-5	2244	Not Detected
	2-6	673	688
	2-7	199	222
	3-3	323	328
XJU-SY	1-3	58	71
	1-4	91	Not Detected
	1-5	21	Not Detected
	2-3	310	319
	2-4	12	12
	2-5	120	130
	3-3	340	352
	3-4	1411	1423
	3-5	6	14

**Table 6:** Network architecture parameters.

Model	Layer Name	Detailed Description
Prosed method	BiLSTM1, BiLSTM2, fully connected layer, regression layer	Number of hidden layer neurons: BiLSTM1 = 93, BiLSTM2 = 90; fully connected layer: layer1 = 45, layer2 = 20; output vector dimension: 1; learning rate: 0.0098; batch size: 128; number of iterations: 2000; dropout rate: 0.15; regression layer = 1
CNN-LSTM	1DConv1, 1DConv1, LSTM, fully connected layer, regression layer	Number of hidden layer neurons: 128; fully connected layer: layer1 = 40; layer2 = 20; output vector dimension: 1; pooling = {1 × 2}; learning rate: 0.001; batch size: 128; number of iterations: 2000; dropout rate: 0.015; regression layer = 1
stacked BiGRU	GRU, Fully connected layer, regression layer	Number of hidden layer neurons: 220; fully connected layer: 45; output vector dimension: 1; learning rate: 0.001; batch size: 128; number of iterations: 2000; dropout rate: 0.15; regression layer = 1

**Table 7:** Time computational complexity and inference latency.

PHM2012			XJU-SY		
Number	SSA Search (s)	Inference Latency (s)	Number	SSA Search (s)	Inference Latency (s)
1-3	2101	4805	1-3	2207	3569
1-4	1879	4652	1-4	2008	3378
1-5	2221	4337	1-5	1995	3487
1-6	1961	4228	2-3	3098	3589
1-7	2004	3879	2-4	3209	2990
2-3	2602	4209	2-5	2111	3244
2-4	2507	3889	3-3	2033	3117
2-5	1995	3809	3-4	2011	3990
2-6	2117	4208	3-5	2207	3876
2-7	2890	4789			
3-3	2368	4602			

**Table 8:** Evaluation metric values.

Proposed Method							
PHM2012				XJU-SY			
Serial Number	RMSE	MAE	R^2^	Serial Number	RMSE	MAE	R^2^
1-3	0.1033	0.0729	0.8720	1-3	0.0630	0.0439	0.9755
1-4	0.0531	0.0390	0.9661	1-4	0.1086	0.1126	0.7320
1-5	0.0978	0.0654	0.7394	1-5	0.1145	0.0855	0.8427
1-6	0.0866	0.0533	0.9100	2-3	0.0446	0.0365	0.9721
1-7	0.0876	0.0709	0.9079	2-4	0.0825	0.0535	0.9432
2-3	0.1167	0.0786	0.8366	2-5	0.0727	0.0523	0.9366
2-4	0.0767	0.0628	0.9294	3-3	0.0516	0.0423	0.9680
2-5	0.0891	0.0648	0.9048	3-4	0.2510	0.2074	0.2437
2-6	0.1316	0.0975	0.7925	3-5	0.1024	0.0780	0.8742
2-7	0.0915	0.0675	0.89960				
3-3	0.0406	0.0296	0.9802				
CNN-BiLSTM							
1-3	0.0503	0.0770	0.8289	1-3	0.0637	0.0440	0.9524
1-4	0.1919	0.1473	−1.0111	1-4	0.1559	0.1286	0.7082
1-5	0.0978	0.0720	0.8808	1-5	0.1339	0.0870	0.8386
1-6	0.0871	0.0567	0.9090	2-3	0.0504	0.0388	0.9696
1-7	0.0876	0.0763	0.4677	2-4	0.0688	0.1236	0.7108
2-3	0.1186	0.2674	0.8312	2-5	0.0826	0.0594	0.9181
2-4	0.0917	0.0638	0.0991	3-3	0.0632	0.0516	−2.788
2-5	0.1356	0.1659	0.6698	3-4	0.2568	0.2199	0.2089
2-6	0.1368	0.1803	0.7753	3-5	0.1075	0.0783	0.8613
2-7	0.5156	0.0763	−2.189				
3-3	0.0532	0.0422	0.9660				
Stacked BiGRU							
1-3	0.0503	0.1733	0.3007	1-3	0.1086	0.0747	0.9513
1-4	0.4094	0.3525	0.0579	1-4	0.1494	0.2607	−0.173
1-5	0.1474	0.0654	0.7394	1-5	0.1160	0.0970	0.7850
1-6	0.1255	0.1054	0.4677	2-3	0.1025	0.0836	0.8738
1-7	0.2106	0.0157	−6.2221	2-4	0.1552	0.0594	0.9184
2-3	0.3346	0.8544	−0.3436	2-5	0.0780	0.0611	0.9271
2-4	0.0782	0.0730	0.9265	3-3	0.5619	0.5347	0.9521
2-5	0.1659	0.0979	0.7794	3-4	0.7627	0.7168	−5.981
2-6	0.2166	0.1004	0.4372	3-5	0.1091	0.0828	0.8572
2-7	0.1192	1.0070	−12.129				
3-3	0.0554	0.0429	0.9632				

## Data Availability

The raw data supporting the conclusions of this article will be made available by the authors on request.
